# Bi-factor and Second-Order Copula Models for Item Response Data

**DOI:** 10.1007/s11336-022-09894-2

**Published:** 2022-11-21

**Authors:** Sayed H. Kadhem, Aristidis K. Nikoloulopoulos

**Affiliations:** School of Computing Sciences, University of East Anglia, Norwich, NR4 7TJ UK

**Keywords:** Bi-factor model, conditional independence, limited information, second-order model, tail dependence/asymmetry, truncated vines

## Abstract

Bi-factor and second-order models based on copulas are proposed for item response data, where the items are sampled from identified subdomains of some larger domain such that there is a homogeneous dependence within each domain. Our general models include the Gaussian bi-factor and second-order models as special cases and can lead to more probability in the joint upper or lower tail compared with the Gaussian bi-factor and second-order models. Details on maximum likelihood estimation of parameters for the bi-factor and second-order copula models are given, as well as model selection and goodness-of-fit techniques. Our general methodology is demonstrated with an extensive simulation study and illustrated for the Toronto Alexithymia Scale. Our studies suggest that there can be a substantial improvement over the Gaussian bi-factor and second-order models both conceptually, as the items can have interpretations of discretized maxima/minima or mixtures of discretized means in comparison with discretized means, and in fit to data.

**Supplementary Information:**

The online version contains supplementary material available at 10.1007/s11336-022-09894-2.

Psychological scales and educational tests are developed to measure a particular construct by selecting items from several identified domains (Gibbons et al. 2007). For example, a questionnaire or instrument, used in psychometrics to assess abstract concepts, such as the well-being has a large number of items or questions that are sampled from several subdomains such as depression, anxiety and stress. This special classification of items in educational assessments is termed ‘testlets’ (Wainer and Kiely 1987). It is essential to investigate the factorial structure, as implementing unstructured factor models on testlet-based items could result in biased estimates and a poor fit (Wang and Wilson 2005; DeMars 2006; Zenisky et al. 2002; Sireci et al. 1991; Lee and Frisbie 1999; Wainer and Thissen 1996).

To account for the homogeneous dependence in several subdomains of some larger domain, Gibbons and Hedeker (1992) and Gibbons et al. (2007) proposed bi-factor models for binary and ordinal items, respectively. They consist of a common factor, that is linked to all items, and non-overlapping group-specific factors. The common factor explains the dependence between all the items, while the group-specific factors explain the dependence amongst items within each domain or group. The items are assumed to be independent given the group-specific and common factors.

An alternative way of modelling items that are split into several domains is via the second-order model (e.g., de la Torre and Song 2009; Rijmen 2010), where items are indirectly mapped to an overall (second-order) factor via non-overlapping group-specific (first-order) factors. Second-order models are suitable when the first-order factors are associated with each other, and there is a second-order factor that accounts for the relations among the first-order factors. The second-order model can be described as an independent clusters factor model (McDonald 1999) with a single second-order factor.

The bi-factor and the second-order models are not generally equivalent (Yung et al. 1999; Gustafsson and Balke 1993; Mulaik and Quartetti 1997; Rijmen 2010), unless proportionality constraints are imposed by using the Schmid–Leiman transformation method (Schmid and Leiman 1957). More importantly, both models are restricted to the MVN assumption for the latent variables, which might not be valid. Nikoloulopoulos and Joe (2015) emphasized that if the ordinal variables in item response can be thought of as discretization of latent random variables that are maxima/minima or mixtures of means, then the use of factor models based on the MVN assumption for the latent variables could provide poor fit. In the context of item response data, latent maxima, minima and means can arise depending on how a respondent considers specific items. An item might make the respondent think about *M* past events which, say, have values $W_1,\ldots ,W_M$. In answering the item, the subject might take the average, maximum or minimum of $W_1,\ldots ,W_M$ and then convert to the ordinal scale depending on the magnitude. The case of a latent maxima/minima can occur if the response is based on a best or worst case.

Nikoloulopoulos and Joe (2015) have studied factor copula models for item response where for the first factor there are bivariate copulas that couple each item to the first latent variable, and for the second factor there are copulas that link each item to the second latent variable conditioned on the first factor (leading to conditional dependence parameters), etc. They have shown that there is an improvement on the factor models based on the MVN assumption for the latent variables both conceptually and in fit to data. This improvement relies on the aforementioned reasons, i.e., items can have more probability in joint upper or lower tail than would be expected with a discretized MVN or items can be considered as discretized maxima/minima or mixtures of discretized means rather than discretized means. When all the bivariate copulas are bivariate normal (BVN), then the resulting model is the same as the discretized MVN model with a *p*-factor correlation matrix (Maydeu-Olivares 2006), also known as the *p*-dimensional normal ogive model (Jöreskog and Moustaki 2001). For example, the 1-factor copula model with BVN copulas is the same as the variant of Samejima’s (1969) graded response IRT model, known as normal ogive model (McDonald 1997) with an 1-factor correlation matrix. We refer to Nikoloulopoulos and Joe (2015, Section 2.3) for further details and explanations on the normal ogive models as special cases of factor copula models.

In this paper, we propose copula extensions for bi-factor and second-order models. The construction of the bi-factor copula model exploits the use of bivariate copulas that link the items to the common and group-specific factors. Note that if there is only one group of items, then the bi-factor model reduces to the two-factor copula model in Nikoloulopoulos and Joe (2015). Similarly with the bi-factor copula model, we also use bivariate copulas to construct the second-order copula model. In this case, there are bivariate copulas that link the items to the group-specific factors, and also bivariate copulas that link the group-specific to the second-order factor. To account for the dependence between the items and group-specific factors, each group of variables in fact is modelled using the one-factor copula model proposed by Nikoloulopoulos and Joe (2015). In addition, if there is only one group of items, then the second-order copula model reduces to the one-factor copula model. Hence, the proposed models contain the one- and two-factor copula models in Nikoloulopoulos and Joe (2015) as special cases, while allowing flexible dependence structure for both within- and between-group dependence. As a result, the models are suitable for modelling a high-dimensional item response classified into non-overlapping groups.

The proposed copula constructions are truncated vine copula models (Brechmann et al. 2012) that involve both observed and latent variables. Joe et al. (2010) have shown that by choosing bivariate linking copulas appropriately, truncated vine copula models can have a wide range of asymmetric dependence as well as tail dependence (dependence among extreme values) and different lower/upper tail dependence parameters for each bivariate margin. Hence, the bi-factor and second-order copula models will be useful when the items have more probability in joint upper or lower tail than would be expected with a discretized MVN. If the bivariate linking copulas are BVN, then the Gaussian bi-factor and second-order models are special cases of our constructions which are the discrete counterparts of the structured factor copula models Krupskii and Joe (2015) where dependence and tail properties are obtained.

The remainder of the paper proceeds as follows: Section 1 introduces the bi-factor and second-order copula models for item response and discusses their relationship with the existing models. Estimation techniques and computational details are provided in Sect. 2. Section 3 proposes simple diagnostics based on semi-correlations and a heuristic method to select suitable bivariate copulas and build plausible bi-factor and second-order copula models. Section 4 summarizes the assessment of goodness of fit of these models using the $M_2$ statistic of Maydeu-Olivares and Joe (2006), which is based on a quadratic form of the deviations of sample and model-based proportions over all bivariate margins. Section 5 contains an extensive simulation study to gauge the small-sample efficiency of the proposed estimation, investigate the misspecification of the bivariate copulas, and examine the reliability of the model selection and goodness-of-fit techniques. Section 6 presents an application of our methodology to the Toronto Alexithymia Scale. In this example, it turns out that our models, with linking copulas selected according to the items being discretized latent minima or mixtures of discretized means, provide better fit than the Gaussian bi-factor and second-order models. We conclude with some discussion in Sect. 7.

## Bi-factor and Second-Order Copula Models

Let $\underbrace{Y_{11}, \ldots ,Y_{d_11}}_1,\ldots , \underbrace{Y_{1g},\ldots , Y_{d_gg}}_g, \ldots , \underbrace{Y_{1G}, \ldots , Y_{d_GG}}_G$ denote the item response variables classified into the *G* non-overlapping groups. There are $d_g$ items in group *g*; $g=1,\ldots ,G,\,j=1,\ldots ,d_g$ and collectively there are $d=\sum _{g=1}^{G} d_g$ items, which are all measured on an ordinal scale; $Y_{jg}\in \{0,\ldots ,K-1\}$. Let the cutpoints in the uniform *U*(0, 1) scale for the *jg*’th item be $a_{jg,k}$, $k=1,\ldots ,K-1$, with $a_{jg,0}=0$ and $a_{jg,K}=1$. These correspond to $a_{jg,k}=\Phi (\alpha _{jg,k})$, where $\alpha _{jg,k}$ are cutpoints in the normal *N*(0, 1) scale.

The bi-factor and second-order factor copula models are presented in Sects. 1.1 and 1.2, respectively. Section 1.3 discusses their relationship with the existing Gaussian bi-factor and second-order models, and Sect. 1.4 provides the bivariate linking copulas we consider along with their properties.

### Bi-factor Copula Model

**Fig. 1:**
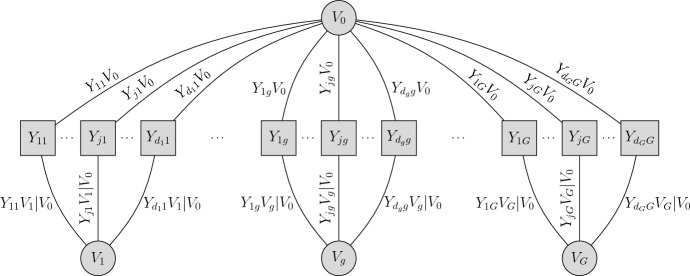
Graphical representation of the bi-factor copula model with *G* group-specific factors and a common factor $V_0$.

Consider a common factor $V_0$ and *G* group-specific factors $V_1,\ldots ,V_G$, where $V_0,V_1,\ldots ,V_G$ are independent and standard uniformly distributed. Let $Y_{jg}$ be the *j*th observed variable in group *g*, with $y_{jg}$ being the realization. The bi-factor model assumes that $Y_{1g},\ldots , Y_{d_gg}$ are conditionally independent given $V_0$ and $V_g$, and that $Y_{jg}$ in group *g* does not depend on $V_{g'}$ for $g\ne g'$. Figure 1 depicts a graphical representation of the model.

The joint probability mass function (pmf) is given by:

$$\begin{aligned} \pi (\textbf{y})= & {} \Pr (Y_{jg} = y_{jg} ; j=1,\ldots ,d_g,g=1,\ldots ,G) \\= & {} \int _{[0,1]^{G+1}} \prod _{g=1}^{G}\prod _{j=1}^{d_g} \Pr (Y_{jg} = y_{jg}| V_0 =v_0, V_g=v_g) d{v_1}\cdots d{v_G} \textrm{d}{v_0}. \end{aligned}$$

According to Sklar’s theorem (1959), there exists a bivariate copula $C_{Y_{jg},V_0}$ such that $\Pr (Y_{jg} \le y_{jg}, V_0 \le v_0)= C_{Y_{jg},V_0}\bigl (F_{{Y_{jg}}}(y_{jg}), v_0\bigr )$, for $v_0 \in [0,1]$, where $C_{Y_{jg},V_0}$ is the copula that links the item $Y_{jg}$ with the common factor $V_0$, $F_{Y_{jg}}$ is the cumulative distribution function (cdf) of $Y_{jg}$; note that $F_{Y_{jg}}$ is a step function with jumps at $0,\ldots ,K-1$, i.e., $F_{Y_{jg}}(y_{jg})=a_{jg,y_{jg}+1}$. Then, it follows that,

$$\begin{aligned} F_{Y_{jg}|V_0}(y_{jg}|v_0):= \Pr (Y_{jg} \le y_{jg} | V_0 = v_0)= \frac{\partial }{\partial v_0} C_{Y_{jg},V_0}\bigl (a_{jg,y_{jg}+1}, v_0\bigr ). \end{aligned}$$

For shorthand notation, we let $C_{Y_{jg}|V_0}\bigl (a_{jg,y_{jg}+1}|v_0\bigr ) = \frac{\partial }{\partial v_0} C_{Y_{jg},V_0}\bigl ( a_{jg,y_{jg}+1}, v_0\bigr )$.

The observed variables also load on the group-specific factors; hence to account for this dependence, we let $C_{Y_{jg},V_g|V_0 }$ be a bivariate copula that links the item $Y_{jg}$ with the group-specific factor $V_g$ given the common factor $V_0$. Hence,

$$\begin{aligned}&\Pr (Y_{jg} \le y_{jg}| V_0 =v_0, V_g=v_g)= \frac{\partial }{\partial v_g} \Pr (Y_{jg} \le y_{jg} , V_g\le v_g | V_0 =v_0) \\&\qquad = \frac{\partial }{\partial v_g} C_{Y_{jg},V_g|V_0}\bigl (F_{Y_jg|V_0}(y_{jg}|v_0), v_g\bigr )= C_{Y_{jg}|V_g;V_0}\bigl (F_{Y_jg|V_0}(y_{jg}|v_0)| v_g\bigr ). \end{aligned}$$

To this end, the pmf of the bi-factor copula model takes the form

1 $$\begin{aligned} \pi (\textbf{y})&= \int _{[0,1]^{G+1}} \prod _{g=1}^{G} \prod _{j=1}^{d_g} \biggr \{C_{Y_{jg}|V_g;V_0}\bigl (F_{Y_{jg}|V_0}(y_{jg}|v_0)| v_g\bigr ) \nonumber \\ {}&\quad -C_{Y_{jg}|V_g;V_0}\bigr (F_{Y_{jg}|V_0}(y_{jg}-1|v_0)| v_g\bigl ) \biggl \} d{v_1}\cdots d{v_G} \textrm{d}{v_0} \nonumber \\&= \int _0^1 \prod _{g=1}^{G} \Bigg \{ \int _0^1 \prod _{j=1}^{d_g} \biggl [ C_{Y_{jg}|V_g;V_0}\bigl (F_{Y_{jg}|V_0}(y_{jg}|v_0)| v_g\bigr )\nonumber \\ {}&\quad -C_{Y_{jg}|V_g;V_0}\bigr (F_{Y_{jg}|V_0}(y_{jg}-1|v_0)| v_g\bigl ) \biggr ] \textrm{d}{v_g} \Bigg \} \textrm{d}{v_0} \nonumber \\&= \int _0^1 \prod _{g=1}^{G} \Bigg \{ \int _0^1 \prod _{j=1}^{d_g} \biggr [ C_{Y_{jg}|V_g;V_0}\bigl ( C_{Y_{jg}|V_0}(a_{jg,y_{jg}+1}|v_0) | v_g\bigr ) \nonumber \\ {}&\quad -C_{Y_{jg}|V_g;V_0}\bigr ( C_{Y_{jg}|V_0}(a_{jg,y_{jg}}|v_0) | v_g\bigr ) \biggl ] \textrm{d}{v_g} \Bigg \} \textrm{d}{v_0}\nonumber \\&= \int _0^1 \prod _{g=1}^{G} \Bigg \{ \int _0^1 \prod _{j=1}^{d_g} f_{Y_{jg}|V_{g};V_0}(y_{jg}|v_g,v_0) \textrm{d}{v_g} \Bigg \} \textrm{d}{v_0}. \end{aligned}$$

It is shown that the pmf is represented as an one-dimensional integral of a function which is in turn a product of *G* one-dimensional integrals. Thus, we avoid $(G+1)$-dimensional numerical integration.

In addition to the computational advancements the proposed model offers, it can provide, with appropriately chosen linking copulas, more probability in joint upper or lower tail than would be expected with a discretized MVN. The bi-factor copula can be explained as a 2-truncated vine. *d*-dimensional vine copulas can cover flexible dependence structures through the specification of *d* bivariate marginal copulas at level 1 and $d(d-1)/2$ bivariate conditional copulas at higher levels (Nikoloulopoulos et al. 2012). For the *d*-dimensional bi-factor copula, the pairs at level 1 are $Y_{jg},V_0$ for $g=1,\ldots ,G,\,j=1,\ldots ,d_g$, the pairs at level 2 are $Y_{jg},V_g|V_0$ for $g=1,\ldots ,G,\,j=1,\ldots ,d_g$, and for higher levels the (conditional) copula pairs are set to independence. That is the bi-factor copula has *d* bivariate copulas $C_{Y_{jg},V_0}$ that link $Y_{jg},\,g=1,\ldots ,G,\,j=1,\ldots ,d_g$ with $V_0$ in the 1st level of the vine, *d* bivariate copulas $C_{Y_{jg},V_g|V_0}$ that link $Y_{jg},\,g=1,\ldots ,G,\,j=1,\ldots ,d_g$ with $V_g,\,g=1,\ldots ,G$ given $V_0$ in the 2nd level of the vine, and independence copulas in all the remaining levels of the vine (truncated after the 2nd level). From results in Joe et al. (2010) and Krupskii and Joe (2015), upper or lower tail dependent copulas in levels 1 and 2 will lead to items that have more probability in joint upper or lower tail than would be expected with a discretized MVN.

For the parametric version of the bi-factor copula model, we let $C_{Y_{jg},V_0}$ and $C_{Y_{jg},V_g|V_0}$ be parametric copulas with dependence parameters $\theta _{jg}$ and $\delta _{jg}$, respectively.

### Second-Order Copula Model

**Fig. 2:**
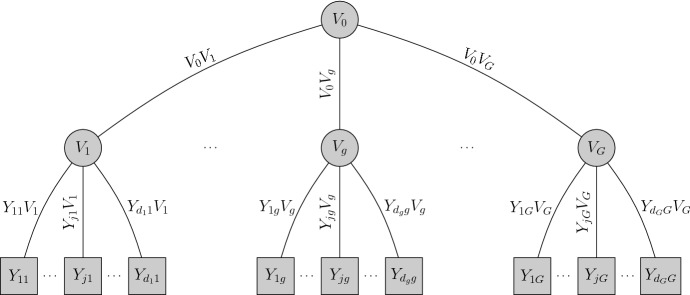
Graphical representation of the second-order copula model with *G* first-order factors and one second-order factor $V_0$.

Assume that for a fixed $g=1,\ldots ,G$, the items $Y_{1g},\ldots , Y_{d_gg}$ are conditionally independent given the first-order factors $V_g \sim U(0,1),\,g=1,\ldots ,G$ and that $\textbf{V}=(V_1,\ldots ,V_G)$ are conditionally independent given the second-order factor $V_0 \sim U(0,1)$. That is the joint distribution of $\textbf{V}$ has an one-factor structure. We also assume that $Y_{jg}$ in group *g* does not depend on $V_{g'}$ for $g\ne g'$. Figure 2 depicts the graphical representation of the model.

The joint pmf takes the form

$$\begin{aligned} \pi (\textbf{y}) =\int _{[0,1]^G} \Bigg \{ \prod _{g=1}^{G} \prod _{j=1}^{d_g} \Pr (Y_{jg} = y_{jg} | V_g = v_g) \Bigg \} c_\textbf{V}(v_1,\ldots , v_G) \textrm{d}{v_1}\cdots \textrm{d}{v_G}; \end{aligned}$$

$c_\textbf{V}$ is the one-factor copula density (Krupskii and Joe 2013) of $\textbf{V}$, viz.

$$\begin{aligned} c_\textbf{V}(v_1,\ldots , v_G) = \int _0^1 \prod _{g=1}^{G} c_{V_g,V_0}(v_g,v_0) \textrm{d}v_0, \end{aligned}$$

where $c_{V_g,V_0}$ is the bivariate copula density of the copula $C_{V_g,V_0}$ linking $V_g$ and $V_0$.

Letting $C_{Y_{jg},V_g}$ be a bivariate copula that joins the item $Y_{jg}$ and the group-specific factor $V_g$ such that

$$\begin{aligned} F_{Y_{jg}|V_g}(y_{jg}|v_g):= \Pr (Y_{jg} \le y_{jg} | V_g = v_g)= \frac{\partial }{\partial v_g} C_{Y_{jg},V_g}\bigl (a_{jg,y_{jg}+1}, v_g\bigr )=C_{Y_{jg}|V_g}\bigl (a_{jg,y_{jg}+1}| v_g\bigr ), \end{aligned}$$

the pmf of the second-order copula model becomes

2 $$\begin{aligned} \pi (\textbf{y})&= \int _0^1 \int _{[0,1]^G} \Bigg \{ \prod _{g=1}^{G} \prod _{j=1}^{d_g} \Big ( C_{Y_{jg}|V_g}\bigl (a_{jg,y_{jg}+1} | v_g\bigr ) - C_{Y_{jg}|V_g}\bigl (a_{jg,y_{jg}} | v_g \bigr ) \Big ) \Bigg \}\nonumber \\ {}&\quad \times \Bigg \{ \prod _{g=1}^{G} c_{V_g,V_0}\bigl (v_g,v_0\bigr ) \Bigg \} \textrm{d}{v_1} \cdots \textrm{d}{v_G} \textrm{d}{v_0}\nonumber \\&= \int _0^1 \Bigg \{ \prod _{g=1}^{G} \int _0^1 \Bigg [ \prod _{j=1}^{d_g} \Big ( C_{Y_{jg}|V_g}\bigl (a_{jg,y_{jg}+1} | v_g\bigr ) - C_{Y_{jg}|V_g}\bigl (a_{jg,y_{jg}} | v_g \bigr ) \Big ) \Bigg ] c_{V_g,V_0}\bigl (v_g,v_0\bigr ) \textrm{d}{v_g} \Bigg \} \textrm{d}{v_0}\nonumber \\&=\int _0^1 \Bigg \{ \prod _{g=1}^{G} \int _0^1 \Big [ \prod _{j=1}^{d_g} f_{Y_{jg}|V_g}(y_{jg}|v_g) \Big ] c_{V_g,V_0}\bigl (v_g,v_0\bigr ) \textrm{d}{v_g} \Bigg \} \textrm{d}{v_0}. \end{aligned}$$

Similarly with the bi-factor copula model, the pmf is represented as an one-dimensional integral of a function, which is in turn a product of *G* one-dimensional integrals.

In addition to the computational advancements, the second-order model offers, it can provide, with appropriately chosen linking copulas, more probability in joint upper or lower tail than would be expected with a discretized MVN. The second-order copula can be explained as an 1-truncated vine. For the *d*-dimensional second-order copula, the pairs at level 1 are $Y_{jg},V_g$ for $g=1,\ldots ,G,\,j=1,\ldots ,d_g$ and $V_0,V_g$ for $g=1,\ldots ,G$, and for higher levels the (conditional) copula pairs are set to independence. That is the second copula has *d* bivariate copulas $C_{Y_{jg},V_g}$ that link $Y_{jg},\,g=1,\ldots ,G,\,j=1,\ldots ,d_g$ with $V_g,\,g=1,\ldots ,G$ and *G* bivariate copulas $C_{V_g,V_0}$ that link $V_{g},\,g=1,\ldots ,G$ with $V_0$ in the 1st level of the vine, and independence copulas in all the remaining levels of the vine (truncated after the 1st level). Joe et al. (2010) have shown that in order for a vine copula to have tail dependence for all bivariate margins, it is only necessary for the bivariate copulas in level 1 to have tail dependence and it is not necessary for the conditional bivariate copulas in levels $2,\ldots ,d$ to have tail dependence. Hence, upper or lower tail dependent copulas in level 1 will lead to will lead to items that have more probability in joint upper or lower tail than would be expected with a discretized MVN.

For the parametric version of the second-order copula model, we let $C_{Y_{jg},V_g}$ and $C_{V_g,V_0}$ be parametric copulas with dependence parameters $\theta _{jg}$ and $\delta _{g}$, respectively.

### Special Cases

In this subsection, we show what happens when all bivariate copulas are BVN. Let $Z_{jg}$ be the underlying continuous variable of the ordinal variable $Y_{jg}$, i.e., $Y_{jg} = y_{jg}$ if $\alpha _{jg,y_{jg}} \le Z_{jg} \le \alpha _{jg,y_{jg}+1}$ with $\alpha _{jg,K} = \infty$ and $\alpha _{jg,0} = -\infty$.

For the bi-factor model, if $C_{Y_{jg},V_0}(\cdot ;\theta _{jg})$ and $C_{Y_{jg},V_g|V_0}(\cdot ;\delta _{jg})$ are BVN copulas,

$$\begin{aligned} C_{Y_{jg}|V_g; V_0}(C_{Y_{jg} | V_0}(a_{jg,y_{jg}+1}| v_0) | v_g) =\Phi \left( \frac{ \alpha _{jg,y_{jg}+1} - \theta _{jg} \Phi ^{-1}(v_0) - \delta _{jg} \sqrt{1-\theta _{jg}^2} \Phi ^{-1}(v_g)}{\sqrt{(1 - \theta _{jg}^2) (1-\delta _{jg}^2) }} \right) . \end{aligned}$$

Hence, the pmf for the bi-factor copula model in (1) becomes:

$$\begin{aligned} \pi (\textbf{y})= & {} \int _0^1 \prod _{g=1}^G \Biggl \{ \int _0^1 \prod _{j=1}^{d_g} \Biggl [ \Phi \left( \frac{ \alpha _{jg,y_{jg}+1} - \theta _{jg} \Phi ^{-1}(v_0) - \delta _{jg} \sqrt{1-\theta _{jg}^2} \Phi ^{-1}(v_g)}{\sqrt{(1 - \theta _{jg}^2) (1-\delta _{jg}^2) }} \right) \\{} & {} -\Phi \left( \frac{ \alpha _{jg,y_{jg}} - \theta _{jg} \Phi ^{-1}(v_0) - \delta _{jg} \sqrt{1-\theta _{jg}^2} \Phi ^{-1}(v_g)}{\sqrt{(1 - \theta _{jg}^2) (1-\delta _{jg}^2) }} \right) \Biggr ] dv_g \Biggr \} \textrm{d}v_0 \\= & {} \int _{-\infty }^{\infty } \prod _{g=1}^G \Biggl \{ \int _{-\infty }^{\infty } \prod _{j=1}^{d_g} \Biggl [ \Phi \left( \frac{ \alpha _{jg,y_{jg}+1} - \theta _{jg} z_{0} - \delta _{jg} \sqrt{1-\theta _{jg}^2} z_{g}}{\sqrt{(1 - \theta _{jg}^2) (1-\delta _{jg}^2) }} \right) \\{} & {} - \Phi \left( \frac{ \alpha _{jg,y_{jg}} - \theta _{jg} z_{0} - \delta _{jg} \sqrt{1-\theta _{jg}^2} z_{g}}{\sqrt{(1 - \theta _{jg}^2) (1-\delta _{jg}^2) }} \right) \Biggr ] \phi (z_{g}) dz_{g} \Biggr \} \phi (z_{0}) \textrm{d}z_{0}. \end{aligned}$$

This model is the same as the Gaussian bi-factor model (Gibbons and Hedeker 1992; Gibbons et al. 2007) with stochastic representation

3 $$\begin{aligned} Z_{jg} = \theta _{jg} Z_{0} + \gamma _{jg} Z_{g} +\sqrt{1 - \theta _{jg}^2 - \gamma _{jg}^2} \epsilon _{jg}, \qquad g=1,\ldots ,G,\quad j=1,\ldots ,d_g, \end{aligned}$$

where $\gamma _{jg}=\delta _{jg}\sqrt{1 - \theta _{jg}^2}$ and $Z_{0},Z_{g},\epsilon _{jg}$ are iid *N*(0, 1) random variables. The parameter $\theta _{jg}$ of $C_{Y_{jg},V_0}$ is the correlation of $Z_{jg}$ and $Z_0$, and the parameter $\delta _{jg}$ of $C_{Y_{jg},V_g|V_0}$ is the partial correlation between $Z_{jg}$ and $Z_{g}=\Phi ^{-1}(V_g)$ given $Z_0=\Phi ^{-1}(V_0)$.

It implies that the underlying random variables $Z_{jg}$’s have a multivariate Gaussian distribution where the off-diagonal entries of the correlation matrix have the form $\theta _{j_1g}\theta _{j_2g} + \gamma _{j_1g}\gamma _{j_2g}$ and $\theta _{j_1g_1}\theta _{j_2g_2}$ for $j_1 \ne j_2$ and $g_1 \ne g_2$, respectively. For the Gaussian bi-factor model to be identifiable, the number of dependence parameters has to be $2d - N_{1}-N_{2}$, where $N_{1}$ and $N_{2}$ is the number of groups that consist of 1 and 2 items, respectively. For a group g of size 1 with variable *j*, $Z_g$ is absorbed with $\epsilon _{jg}$ because $\gamma _{jg}$ would not be identifiable. For a group *g* of size 2 with variable indices $j_1,j_2$, the parameters $\gamma _{j_1g}$ and $\gamma _{j_2g}$ appear only in one correlation; hence, one of $\gamma _{j_1g},\gamma _{j_2g}$ can be taken as 1 without loss of generality. For the bi-factor copula with non-Gaussian linking copulas, near non-identifiability can occur when there are groups of size 2; in this case, one of the linking copulas to the group latent variable can be fixed at comonotonicity.

For the Gaussian second-order model, let $Z_0,Z_1',\ldots ,Z_G'$ be the dependent latent *N*(0, 1) variables, where $Z_0$ is the second-order factor and $Z_g'=\beta _{g}Z_0+\sqrt{1-\beta _{g}^2}Z_g$ is the first-order factor for group *g*. That is, there is an one second-order factor $Z_0$, and the first-order factors $Z_1',\ldots ,Z_G'$ are linear combinations of the second-order factor, plus a unique variable $Z_g$ for each first-order factor. The stochastic representation is (Krupskii and Joe 2015):

$$\begin{aligned} Z_{jg}= & {} \beta _{jg} Z _g' +\sqrt{1-\beta _{jg}^2}\epsilon _{jg}\\ Z_g'= & {} \beta _{g}Z_0+\sqrt{1-\beta _{g}^2}Z_g, \qquad g=1,\ldots ,G, \quad j=1,\ldots ,d_g, \end{aligned}$$

or

4 $$\begin{aligned} Z_{jg}=\beta _{jg} \beta _{g}Z_0+\beta _{jg}\sqrt{1-\beta _{g}^2}Z_g +\sqrt{1-\beta _{jg}^2}\epsilon _{jg}, g=1,\ldots ,G, \quad j=1,\ldots ,d_g. \end{aligned}$$

Hence, this is a special case of (3) where $\theta _{jg} = \beta _{jg} \beta _{g}$ and $\gamma _{jg} = \beta _{jg} \sqrt{1-\beta _{g}^2}$.

### Other Choices of Parametric Bivariate Copulas

In line with Nikoloulopoulos and Joe (2015), we use bivariate parametric copulas that can be used when considering latent maxima, minima or mixtures of means. For different dependent items based on latent maxima or minima, multivariate extreme value and copula theory (e.g., Joe 1997) can be used to select suitable copulas that link observed to latent variables. Copulas that arise from extreme value theory have more probability in one joint tail (upper or lower) than expected with a discretized MVN distribution or a MVN copula with discrete margins. If item responses are based on discretizations of latent variables that are means, then it is possible that there can be more probability in both the joint upper and joint lower tail, compared with discretized MVN models. This happens if the respondents consist of a ‘mixture’ population (e.g., different locations or genders). From the theory of elliptical distributions and copulas (e.g., McNeil et al. 2005), it is known that the multivariate Student-*t* distribution as a scale mixture of MVN has more dependence in the tails. Extreme value and elliptical copulas can model item response data that have reflection asymmetric and symmetric dependence, respectively.

A bivariate copula *C* is reflection symmetric if its density satisfies $c(u_1,u_2)=c(1-u_1,1-u_2)$ for all $0\le u_1,u_2\le 1$. Otherwise, it is reflection asymmetric often with more probability in the joint upper tail or joint lower tail. Upper tail dependence means that $c(1-u,1-u)=O(u^{-1})$ as $u\rightarrow 0$ and lower tail dependence means that $c(u,u)=O(u^{-1})$ as $u\rightarrow 0$. If $(U_1,U_2)\sim C$ for a bivariate copula *C*, then $(1-U_1,1-U_2)\sim \widehat{C}$, where $\widehat{C}(u_1,u_2)=u_1+u_2-1+C(1-u_1,1-u_2)$ is the survival or reflected copula of *C*; this “reflection” of each uniform *U*(0, 1) random variable about 1/2 changes the direction of tail asymmetry.

After briefly providing definitions of tail dependence and reflection symmetry/asymmetry, we provide below the bivariate copula choices we consider:

- The extreme value Gumbel copula with cdf
  $$\begin{aligned} C(u_1,u_2;\theta )=\exp \Bigl [-\Bigl \{(-\log u_1)^{\theta } +(-\log u_2)^{\theta }\Bigr \}^{1/\theta }\Bigr ], \theta \ge 1. \end{aligned}$$
  A model with bivariate Gumbel copulas has latent (ordinal) variables that can be considered as (discretized) maxima, and there is more probability in the joint upper tail as the Gumbel copula has reflection asymmetry and upper tail dependence.
- The survival Gumbel (s.Gumbel) copula with cdf
  $$\begin{aligned} C(u_1,u_2;\theta )= & {} u_1+u_2-1 \\ {}{} & {} + \exp \Bigl [-\Bigl \{\bigl (-\log (1-u_1)\bigr )^{\theta } +\bigl (-\log (1-u_2)\bigr )^{\theta }\Bigr \}^{1/\theta }\Bigr ], \theta \ge 1. \end{aligned}$$
  A model with bivariate s.Gumbel copulas has latent (ordinal) variables that can be considered as (discretized) minima, and there is more probability in the joint lower tail as the s.Gumbel copula has reflection asymmetry and lower tail dependence.
- The elliptical bivariate $t_\nu$ copula with cdf
  $$\begin{aligned} C(u_1,u_2;\theta )=\mathcal {T}_2\Bigl (\mathcal {T}^{-1}(u_1;\nu ),\mathcal {T}^{-1}(u_2;\nu );\theta ,\nu \Bigr ),-1\le \theta \le 1, \end{aligned}$$
  where $\mathcal {T}(;\nu )$ is the univariate Student t cdf with (non-integer) $\nu$ degrees of freedom, and $\mathcal {T}_2$ is the cdf of a bivariate Student-*t* distribution with $\nu$ degrees of freedom and correlation parameter $\theta$. A model with bivariate $t_\nu$ copulas has latent (ordinal) variables that can be considered as mixtures of (discretized) means, since the bivariate Student-*t* distribution arises as a scale mixture of bivariate normals. A small value of $\nu$, such as $1 \le \nu \le 5$, leads to a model with more probabilities in the joint upper and joint lower tails compared with the BVN copula as the $t_\nu$ copula has reflection symmetric upper and lower tail dependence.

The BVN and $t_\nu$ are comprehensive copulas, i.e., they interpolate between countermonotonicity (perfect negative dependence) to comonotonicity (perfect positive dependence), but the Gumbel copulas interpolates between independence and perfect positive dependence. Nevertheless, negative dependence or interpolation between perfect negative dependence and independence can be obtained from the Gumbel copulas by considering reflection of one of the uniform random variables on (0, 1). If $(U_1,U_2)\sim C$ for a bivariate copula *C* with positive dependence, then

- $(1-U_1,U_2)\sim \widehat{C}^{(1)}$, where $\widehat{C}^{(1)}(u_1,u_2)=u_2-C(1-u_1,u_2)$ is the 1-reflected copula of *C* with negative lower-upper tail dependence;
- $(U_1,1-U_2)\sim \widehat{C}^{(2)}$, where $\widehat{C}^{(2)}(u_1,u_2)=u_1-C(u_1,1-u_2)$ is the 2-reflected copula of *C* with negative upper-lower dependence.

Negative upper-lower tail dependence means that $c(1-u,u)=O(u^{-1})$ as $u\rightarrow 0^+$ and negative lower-upper tail dependence means that $c(u,1-u)=O(u^{-1})$ as $u\rightarrow 0^+$ (Joe 2011). The proposed models can provide, with linking copulas that allow for negative (tail) dependence, negative (tail) dependence between the observed variables as the dependence between the observed and latent variables is inherited to the dependence amongst the observed variables.

**Fig. 3:**
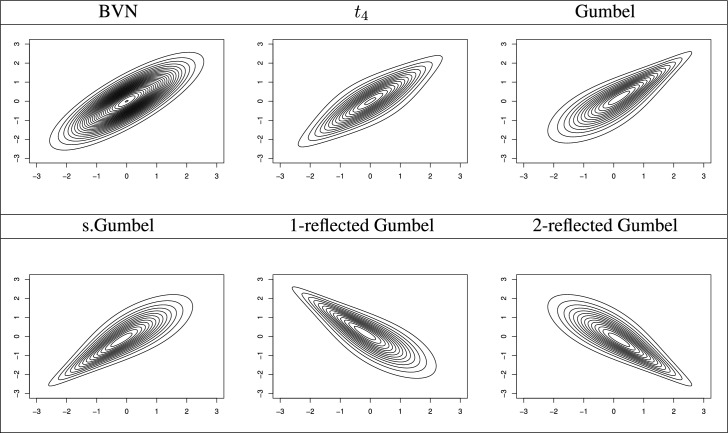
Contour plots of bivariate copulas with standard normal margins and dependence parameters corresponding to Kendall’s $\tau$ value of 0.6 in absolute value.

In Fig. 3, to depict the concepts of refection symmetric or asymmetric tail dependence, we show contour plots of the corresponding copula densities with standard normal margins and dependence parameters corresponding to Kendall’s $\tau$ value of 0.6 in absolute value. To make the models comparable, we convert the BVN/$t_\nu$ and (reflected) Gumbel copula parameters to Kendall’s $\tau$’s via

5 $$\begin{aligned} \tau (\theta )=\frac{2}{\pi }\arcsin (\theta ) \end{aligned}$$

and

6 $$\begin{aligned} \quad \tau (\theta )=\left\{ \begin{array}{lll}1-\theta ^{-1},&{}\quad C, \widehat{C}\\ \theta ^{-1}-1,&{}\quad \widehat{C}^{(1)},\widehat{C}^{(2)}\end{array}\right. , \end{aligned}$$

respectively. Sharper corners (relative to ellipse) in Fig. 3 indicate tail dependence.

The Kendall’s tau parameters, which are strictly increasing functions of the copula parameters, account for the dependence dominated by the middle of the data, and they are expected to be similar among different families of bivariate copulas. However, the tail dependence varies and it is a property to consider when choosing among different families of bivariate copulas (Nikoloulopoulos and Karlis 2008).

## Estimation and Computational Details

For the set of all parameters, let $\varvec{\theta }=(\textbf{a},\varvec{\theta }_g,\varvec{\delta }_{g})$ for the bi-factor copula model and $\varvec{\theta }=(\textbf{a},\varvec{\theta }_g,\varvec{\delta })$ for the second-order copula model, where $\textbf{a}=(a_{jg,k}: j=1,\ldots ,d_g, g=1,\ldots ,G, k=1,\ldots ,K-1)$, $\varvec{\theta }_g=(\theta _{1g}, \ldots , \theta _{jg}, \ldots ,\theta _{d_gg}: g=1,\ldots ,G)$, $\varvec{\delta }_{g}=(\delta _{1g}, \ldots , \delta _{jg}, \ldots ,\delta _{d_gg}: g=1,\ldots ,G)$ and $\varvec{\delta }=(\delta _{1}, \ldots ,\delta _{G})$. The dimension of $\textbf{a}$, $\varvec{\theta }_g$, $\varvec{\delta }_{g}$ and $\varvec{\delta }$ are $d(K-1)$, *d*, *d* and G, respectively. Hence, the dimension *q* of $\varvec{\theta }$ is $d(K+1)$ and $dK +G$ for the bi-factor and second-order copula model, respectively.

With sample size *n* and data $\textbf{y}_1,\ldots ,\textbf{y}_n$, the joint log-likelihood of the bi-factor and second-order copula is

7 $$\begin{aligned} \ell (\varvec{\theta };\textbf{y}_1,\ldots ,\textbf{y}_n)=\sum _{i=1}^n\log \pi (\textbf{y}_i;\varvec{\theta }). \end{aligned}$$

with $\pi (\textbf{y}_i;\varvec{\theta })$ as in (1) and (2), respectively. Maximum likelihood (ML) estimation, i.e., maximization of (7), is numerically possible but time-consuming for large *d* because the large number of univariate cutpoints and dependence parameters. Hence, we approach estimation using the two-step Inference Function of Margins (IFM) method (Joe and Xu 1996; Joe 1997). Joe (2005) has established its asymptotic efficiency and has shown that can efficiently, in the sense of computing time and asymptotic variance, estimate the univariate and dependence parameters.

In the first step of the IFM, the univariate parameters, i.e., the cutpoints, are estimated using the univariate sample proportions. The univariate cutpoints for the *j*th item in group *g* are estimated as $\hat{a}_{jg,k} = \sum _{y=0}^{k} p_{jg,y}$, where $p_{jg,y}\,,y=0,\ldots ,K-1$ for $g=1,\ldots ,G$ and $j=1,\ldots ,d_g$ are the univariate sample proportions. In the second step of the IFM method, the joint log-likelihood in (7) is maximized over the copula parameters with the cutpoints fixed as estimated at the first step. That is for $i=1,\ldots ,n$ we start from a *d*-variate sample $y_{i11}, \ldots ,y_{id_11},\ldots , y_{i1G}, \ldots , y_{id_GG}$ from which *d* estimators $F_{11}(y_{i11}), \ldots ,F_{d_11}(y_{id_11}), \ldots , F_{1G}(y_{i1G}), \ldots ,$ $F_{d_GG}(y_{id_GG})$ are obtained. We use these estimators, i.e., the cutpoints, to transform the $y_{i11}, \ldots ,y_{id_11},\ldots , y_{i1G},$ $\ldots , y_{id_GG}$ sample to a uniform sample ${\hat{\alpha }}_{11,y_{i11}+1},\ldots ,{\hat{\alpha }}_{d_11,y_{id_11}+1},\ldots {\hat{\alpha }}_{1G,y_{i1G}+1}, \ldots , {\hat{\alpha }}_{d_GG,y_{id_GG}+1}$ on $[0, 1]^d$ and then fit the factor copula model at the second step. Hence, the IFM approach can be regarded as a two-step approach on the original data or simply as the standard one-step ML method on the transformed (copula) data. Note also in passing that compared to the ML, the IFM method is not as punishing for misspecification of the dependence structure (Joe and Xu 1996; Xu 1996).

For the bi-factor copula model, numerical evaluation of the joint pmf can be achieved with the following steps:

1. Calculate Gauss–Legendre quadrature (Stroud and Secrest 1966) points $\{\mathcal {v}_q: q=1,\ldots ,n_q\}$ and weights $\{w_q: q=1,\ldots ,n_q\}$ in terms of standard uniform.
2. Numerically evaluate the joint pmf
  $$\begin{aligned} \int _0^1 \prod _{g=1}^{G} \Bigg \{ \int _0^1 \prod _{j=1}^{d_g} f_{Y_{jg}|V_{jg};V_0}(y_{jg}|v_g,v_0) \textrm{d}{v_g} \Bigg \} \textrm{d}{v_0} \end{aligned}$$
  in a double sum
  $$\begin{aligned} \sum _{q_1=1}^{n_q} w_{q_1} \prod _{g=1}^{G} \Bigg \{ \sum _{q_2=1}^{n_q} w_{q_2} \prod _{j=1}^{d_g} f_{Y_{jg}|V_{jg};V_0}(y_{jg}|\mathcal {v}_{q_2},\mathcal {v}_{q_1}) \Bigg \}. \end{aligned}$$

For the second-order copula model, numerical evaluation of the joint pmf can be achieved with the following steps:

1. Calculate Gauss–Legendre quadrature points $\{\mathcal {v}_q : q=1,\ldots , n_q \}$ and weights $\{w_q : q=1,\ldots , n_q \}$ in terms of standard uniform.
2. Numerically evaluate the joint pmf
  $$\begin{aligned} \int _0^1 \Bigg \{ \prod _{g=1}^{G} \int _0^1 \Big [ \prod _{j=1}^{d_g} f_{Y_{jg}|V_g}(y_{jg}|v_g;\theta _{jg}) \Big ] c_{V_g,V_0}\bigl (v_g,v_0;\delta _{g}\bigr ) \textrm{d}{v_g} \Bigg \} \textrm{d}{v_0} \end{aligned}$$
  in a double sum
  $$\begin{aligned} \sum _{q_1=1}^{n_q} w_{q_1} \Bigg \{ \prod _{g=1}^{G} \sum _{q_2=1}^{n_q} w_{q_2} \Big [ \prod _{j=1}^{d_g} f_{Y_{jg}|V_g}(y_{jg}|\mathcal {v}_{q_2|q_1};\theta _{jg}) \Big ] \Bigg \}, \end{aligned}$$
  where $\mathcal {v}_{q_2|q_1} = C^{-1}_{Y_{jg}|V_g;V_0}( {v}_{q_2} | {v}_{q_1};\delta _{g})$. Note that the independent quadrature points $\{\mathcal {v}_{q_1}: q_1 = 1,\ldots ,n_q\}$ and $\{\mathcal {v}_{q_2}: q_2 = 1,\ldots ,n_q\}$ have been converted to dependent quadrature points that have an one-factor copula distribution $C_{X}(\cdot ;\varvec{\delta })$.

The estimated copula parameters can be obtained by using a quasi-Newton (Nash 1990) method applied to the logarithm of the joint likelihood. With Gauss–Legendre quadrature, the same nodes and weights are used for different functions; this helps in yielding smooth numerical derivatives for numerical optimization via quasi-Newton. Our comparisons show that $n_q=25$ quadrature points are adequate with good precision.

## Bivariate Copula Selection

In the following subsections, we describe simple diagnostics based on semi-correlations and a heuristic method that automatically selects the bivariate parametric copula families that build either the bi-factor or the second-order copula model.

Choices of copulas with upper or lower tail dependence are better if the items have more probability in joint lower or upper tail than would be expected with the BVN copula. This can be shown with summaries of polychoric correlations in the upper and lower joint tail (Kadhem and Nikoloulopoulos 2021). In the context of items that can be split into *G* non-overlapping groups, such that there is homogeneous dependence within each group, it is sufficient to (a) summarize the average of the polychoric semi-correlations for all pairs within each of the *G* groups and for all pairs of items, and (b) not mix bivariate copulas for a single factor; hence, for both the bi-factor and second-order copula models we allow $G+1$ different copula families, one for each group specific factor $V_g$ and one for $V_0$.

We distinguish the simple descriptives, i.e., the semi-correlations, from the heuristic algorithm and model fitting. On the one hand, the descriptive statistics can suggest more probability to the lower or upper tail for many pairs of items, but bi-factor and second-order copula models with asymmetrical dependence can be used to check whether the two tails are significantly different.

### Simple Diagnostics Based on Semi-correlations

Consider again the underlying *N*(0, 1) latent variables $Z_{jg}$’s of the ordinal variables $Y_{jg}$’s. The correlations of $Z_{jg}$’s in the upper and lower tail, hereafter semi-correlations, are defined as (Joe 2014, p. 71):

8 $$\begin{aligned} \rho _N^+= & {} \text{ Cor }\Bigl (Z_{jg},Z_{j'g'}|Z_{jg}>0,Z_{j'g'}>0\Bigr )\\= & {} \frac{\int _{0}^{\infty }\int _{0}^{\infty } z_1z_2\phi (z_1)\phi (z_2)c\bigl (\Phi (z_1),\Phi (z_2)\bigr )dz_1dz_2-{\biggr (\int _0^\infty z\phi (z)\Bigr (1- C_{2|1}\bigr (0.5|\Phi (z)\bigl )\Bigr )dz\biggr )^2}/{C(0.5,0.5)}}{\int _0^\infty z^2\phi (z)\Bigr (1- C_{2|1}\bigr (0.5|\Phi (z)\bigl )\Bigr )\textrm{d}z-{\biggr (\int _0^\infty z\phi (z)\Bigr (1- C_{2|1}\bigr (0.5|\Phi (z)\bigl )\Bigr )\textrm{d}z\biggr )^2}/{C(0.5,0.5)}};\nonumber \\ \rho _N^-= & {} \text{ Cor }\Bigl (Z_{j_1g},Z_{j_2g}|Z_{j_1g}<0,Z_{j_2g}<0\Bigr )\nonumber \\= & {} \frac{\int _{-\infty }^{0}\int _{-\infty }^{0} z_1z_2\phi (z_1)\phi (z_2)c\bigl (\Phi (z_1),\Phi (z_2)\bigr )\textrm{d}z_1\textrm{d}z_2-{\biggr (\int _{-\infty }^0 z\phi (z)C_{2|1}\bigr (0.5|\Phi (z)\bigl )\textrm{d}z\biggr )^2}/{C(0.5,0.5)}}{\int _{-\infty }^0 z^2\phi (z) C_{2|1}\bigr (0.5|\Phi (z)\bigl )dz-{\biggr (\int _{-\infty }^0 z\phi (z)C_{2|1}\bigr (0.5|\Phi (z)\bigl )\textrm{d}z\biggr )^2}/{C(0.5,0.5)}}.\nonumber \end{aligned}$$

From the above expressions, it is clear that the semi-correlations depend only on the copula C of $\Bigl (\Phi (Z_{jg}),$ $\Phi (Z_{j'g'})\Bigr )$; $C_{2|1}$ is the conditional copula cdf. For the BVN and $t_\nu$ copulas $\rho _N^{-}=\rho _N^{+}$, while for the Gumbel and s.Gumbel copulas $\rho _N^{-}<\rho _N^{+}$ and $\rho _N^{-}>\rho _N^{+}$, respectively. The sample versions of $\rho _N^{+},\rho _N^{-}$ for item response data are the polychoric correlations in the joint lower and upper quadrants of $Y_{jg}$ and $Y_{j'g'}$ (Kadhem and Nikoloulopoulos 2021).

### Selection Algorithm

We propose a heuristic method that selects appropriate bivariate copulas for each factor of the bi-factor and second-order copula models. It starts with an initial assumption, that all bivariate linking copulas are BVN copulas, i.e. the starting model is either the Gaussian bi-factor or second-order model, and then, sequentially other copulas with lower or upper tail dependence are assigned to the factors where necessary to account for more probability in one or both joint tails. The selection algorithm involves the following steps:

1. Fit the bi-factor or second-order copula model with BVN copulas.
2. Fit all the possible bi-factor or second-order copula models, iterating over all the copula candidates that link all items $Y_{jg}$’s in group *g* or each group-specific factor $V_g$, respectively, to $V_0$.
3. Select the copula family that corresponds to the lowest Akaike information criterion (AIC), that is, $\text {AIC}= -2 \times \ell +2 \times \#\text {copula parameters}$.
4. Fix the selected copula family that links the observed (bi-factor model) or latent (second-order model) variables to $V_0$.
5. For $$g=1,\dots ,G$$:
  - Fit all the possible models, iterating over all the copula candidates that link all the items in group *g* to the group-specific factor $V_g$.
  - Select the copula family that corresponds to the lowest AIC.
  - Fix the selected linking copula family for all the items in group *g* with $V_g$.

For vine copula models (bi-factor and second-order copula models are vine copula models that involve both observed and latent variables), Dissmann et al. (2013) also found that bivariate copula selection based on AIC seems to be better than even using bivariate goodness-of-fit tests. The goodness-of-fit procedures involve a global distance measure between the model-based and empirical distribution; hence, they might not be sensitive to tail behaviours and are not diagnostics in the sense of suggesting improved parametric models in the case of small *p*-values (Joe 2014, p. 254). A smaller AIC indicates a model that better approximates both the dependence structure of the data and the strength of dependence in the tails.

## Goodness of Fit

We will use the limited information $M_2$ statistic proposed by Maydeu-Olivares and Joe (2006) to evaluate the overall fit of the proposed bi-factor and second-order copula models. The $M_2$ statistic is based on a quadratic form of the deviations of sample and model-based proportions over all bivariate margins. For the bi-factor and second-order copula models with parameter vector $\varvec{\theta }$ of dimension *q*, let $\varvec{\pi }_2(\varvec{\theta })=\bigl (\dot{\varvec{\pi }}_1(\varvec{\theta })^\top ,\dot{\varvec{\pi }}_2(\varvec{\theta })^\top \bigr )^\top$ be the column vector of the univariate and bivariate model-based marginal probabilities that do not include category 0 with sample counterpart $\textbf{p}_2=(\dot{\textbf{p}}_1^\top ,\dot{\textbf{p}}_2^\top )^\top$. The total number of the univariate and bivariate residuals $\bigl (\textbf{p}_2-\varvec{\pi }_2({\hat{\varvec{\theta }}})\bigr )^\top$ is

$$\begin{aligned} s = d (K-1) + \left( {\begin{array}{c}d\\ 2\end{array}}\right) (K - 1)^2, \end{aligned}$$

where $d (K-1)$ is the dimension of the univariate residuals and $$\left( {\begin{array}{c}d\\ 2\end{array}}\right) (K - 1)^2$$ is the dimension of the bivariate residuals excluding category 0.

With a sample size *n*, the limited-information $M_2$ statistic is given by

$$\begin{aligned} M_2=M_2({\hat{\varvec{\theta }}})=n\bigl (\textbf{p}_2-\varvec{\pi }_2({\hat{\varvec{\theta }}})\bigr )^\top \textbf{C}_2({\hat{\varvec{\theta }}})\bigl (\textbf{p}_2-\varvec{\pi }_2\bigl ({\hat{\varvec{\theta }}})\bigr ), \end{aligned}$$

with

$$\begin{aligned} \textbf{C}_2(\varvec{\theta })=\varvec{\Xi }_2^{-1}-\varvec{\Xi }_2^{-1}\varvec{\Delta }_2(\varvec{\Delta }_2^\top \varvec{\Xi }_2^{-1}\varvec{\Delta }_2)^{-1}\varvec{\Delta }_2^\top \varvec{\Xi }_2^{-1} =\varvec{\Delta }_2^{(c)}\bigl ([\varvec{\Delta }_2^{(c)}]^\top \varvec{\Xi }_2\varvec{\Delta }_2^{(c)}\bigr )^{-1}[\varvec{\Delta }_2^{(c)}]^\top , \end{aligned}$$

where $\varvec{\Delta }_2=\partial \varvec{\pi }_2(\varvec{\theta })/\partial \varvec{\theta }^\top$ is an $s\times q$ matrix of full column rank *q* with the first-order derivatives of the univariate and bivariate marginal probabilities with respect to the estimated model parameters (in the Supplementary Tables 1–5 of the electronic supplementary material, we provide details on the calculation of these derivatives), $\varvec{\Delta }_2^{(c)}$ is an $s\times (s-q)$ orthogonal complement to $\varvec{\Delta }_2$ of full column rank $s-q$, such that $[\varvec{\Delta }_2^{(c)}]^\top \varvec{\Delta }_2=\textbf{0}$ and $\varvec{\Xi }_2$ is the asymptotic $s \times s$ covariance matrix of $\sqrt{n}\bigl (\textbf{p}_2-\varvec{\pi }_2({\hat{\varvec{\theta }}})\bigr )^\top$.

The asymptotic covariance matrix $\varvec{\Xi }_2$ can be partitioned according to the partitioning of $\textbf{p}_2$ into $\varvec{\Xi }_{11}=\sqrt{n}\text{ Acov }(\dot{\textbf{p}}_1)$, $\varvec{\Xi }_{21}=\sqrt{n}\text{ Acov }(\dot{\textbf{p}}_2,\dot{\textbf{p}}_1)$ and $\varvec{\Xi }_{22}=\sqrt{n}\text{ Acov }(\dot{\textbf{p}}_2)$, where $\text{ Acov }(\cdot )$ denotes asymptotic covariance matrix. The elements of $\varvec{\Xi }_{11}$, $\varvec{\Xi }_{21}$ and $\varvec{\Xi }_{22}$ involve up to the 4-dimensional probabilities as shown below:

$$\begin{aligned}{} & {} \sqrt{n}\text{ Acov }(p_{j_1,y_1},p_{j_2,y_2})=\pi _{j_1j_2,y_1y_2}-\pi _{j_1,y_1}\pi _{j_2,y_2}\\{} & {} \sqrt{n}\text{ Acov }(p_{j_1j_2,y_1y_2},p_{j_3,y_3})=\pi _{j_1j_2j_3,y_1y_2y_3}-\pi _{j_1j_2,y_1y_2}\pi _{j_3,y_3}\\{} & {} \sqrt{n}\text{ Acov }(p_{j_1j_2,y_1y_2},p_{j_3j_4,y_3y_4})=\pi _{j_1j_2j_3j_4,y_1y_2y_3y_4}-\pi _{j_1j_2,y_1y_2}\pi _{j_3j_4,y_3y_4}, \end{aligned}$$

where $$ \pi _{j,y} = \Pr (Y_j = y)$$, $$\pi _{j_1j_2,y_1y_2}= \Pr (Y_{j_1} = y_1,Y_{j_2} = y_2)$$, $$\pi _{j_1j_2j_3,y_1y_2y_3}=\Pr (Y_{j_1} = y_1,Y_{j_2} = y_2,Y_{j_3} = y_3)$$, and $$\pi _{j_1j_2j_3j_4,y_1y_2y_3y_4}=\Pr (Y_{j_1} = y_1,Y_{j_2} = y_2,Y_{j_3} = y_3,Y_{j_4} = y_4)$$.

The limited information statistic $M_2$ under the null hypothesis has an asymptotic distribution that is $\chi ^2$ with $s-q$ degrees of freedom as the IFM estimate ${\hat{\varvec{\theta }}}$ is $\sqrt{n}$-consistent.

## Simulations

An extensive simulation study is conducted to (a) gauge the small-sample efficiency of the IFM estimation method and investigate the misspecification of the bivariate pair-copulas, (b) examine the reliability of using the heuristic algorithm to select the true (simulated) bivariate linking copulas, and (c) study the small-sample performance of the $M_2$ statistic.

**Table 1:** Small sample of size $n = 500$ simulations (10$^3$ replications) from the bi-factor and second-order copula models with Gumbel copulas and group estimated average biases, root mean square errors (RMSE), and standard deviations (SD), scaled by *n*, for the IFM estimates under different pair-copulas from the bi-factor and second-order copula models.

		Bi-factor copula model								Second-order copula model							
		$\tau (\varvec{\theta }_g),\,g=1,\dots ,4$				$\tau (\varvec{\delta }_g),\,g=1,\dots ,4$				$\tau (\varvec{\delta })$				$\tau (\varvec{\theta }_g),\,g=1,\dots ,4$			
	K	0.45	0.55	0.65	0.75	0.30	0.35	0.40	0.50	0.30	0.35	0.40	0.45	0.40	0.50	0.60	0.70
*nbias*																	
BVN	3	2.65	2.54	2.66	2.16	6.60	7.81	6.99	6.39	5.58	5.34	5.33	5.60	0.41	0.86	0.62	0.27
	5	1.98	2.27	2.54	2.53	5.99	6.27	5.42	2.31	8.71	8.36	7.94	8.52	0.93	0.51	0.58	2.52
Gumbel	3	0.39	0.35	0.28	0.34	0.89	1.02	1.62	3.40	$-$ 0.18	0.18	0.18	1.88	0.22	0.67	1.14	2.37
	5	0.23	0.23	0.07	0.20	0.84	0.85	1.02	1.98	0.22	0.13	$-$ 0.25	1.15	0.23	0.43	0.63	0.60
s.Gumbel	3	3.59	3.03	1.51	0.31	4.86	4.52	4.21	1.19	18.43	18.29	18.54	18.68	6.32	6.18	5.47	3.67
	5	0.79	2.25	3.80	5.30	15.89	15.82	13.89	14.52	25.65	24.80	23.58	22.59	3.77	2.54	1.24	2.74
$t_5$	3	1.65	2.81	3.28	3.48	6.99	8.20	7.07	4.89	7.98	8.55	9.18	9.55	3.36	3.56	4.71	3.81
	5	0.49	0.49	0.84	0.92	5.81	6.09	5.58	1.69	9.71	10.05	9.82	9.87	2.24	2.29	2.64	0.36
*nSE*																	
BVN	3	15.03	13.42	12.37	11.06	30.77	31.20	33.07	39.93	22.80	24.94	24.97	27.03	16.82	16.41	17.06	21.32
	5	13.68	11.89	10.63	8.95	24.58	25.33	25.70	29.86	21.28	23.04	22.45	24.72	15.09	14.27	14.01	15.41
Gumbel	3	15.10	13.81	12.33	10.97	29.61	31.34	32.82	42.17	22.58	24.73	25.35	27.87	16.99	16.73	17.66	22.02
	5	13.67	12.29	10.55	8.76	23.60	24.72	25.39	31.13	20.75	22.75	22.69	24.86	15.31	14.62	14.33	15.72
s.Gumbel	3	15.58	13.76	12.60	11.27	33.77	34.80	38.18	51.31	25.34	26.80	27.19	29.36	17.40	16.49	16.59	18.46
	5	14.11	12.30	11.16	9.66	27.08	28.44	30.18	40.10	22.61	24.13	23.36	25.46	15.90	14.57	14.38	16.89
$t_5$	3	15.29	13.54	12.27	10.79	31.43	31.74	33.02	39.02	23.59	25.57	25.65	27.61	17.48	16.69	17.64	22.03
	5	13.84	11.99	10.55	8.80	24.79	25.35	25.66	29.10	21.67	23.52	22.67	24.52	15.40	14.52	14.03	14.88
*nRMSE*																	
BVN	3	15.28	13.66	12.66	11.27	31.48	32.19	33.81	40.45	23.47	25.50	25.53	27.60	16.83	16.44	17.08	21.33
	5	13.83	12.11	10.93	9.30	25.31	26.10	26.27	29.96	22.99	24.51	23.81	26.14	15.12	14.28	14.03	15.62
Gumbel	3	15.10	13.81	12.34	10.98	29.63	31.37	32.87	42.31	22.58	24.73	25.35	27.94	16.99	16.75	17.71	22.15
	5	13.67	12.30	10.55	8.77	23.62	24.74	25.42	31.20	20.75	22.75	22.69	24.88	15.31	14.63	14.35	15.73
s.Gumbel	3	16.00	14.09	12.69	11.27	34.13	35.13	38.42	51.33	31.33	32.45	32.91	34.80	18.52	17.65	17.49	18.82
	5	14.14	12.51	11.79	11.02	31.41	32.55	33.22	42.67	34.19	34.60	33.19	34.04	16.35	14.82	14.44	17.13
$t_5$	3	15.40	13.83	12.71	11.34	32.21	32.80	33.77	39.32	24.91	26.97	27.24	29.21	17.80	17.08	18.27	22.36
	5	13.85	12.01	10.59	8.86	25.47	26.08	26.26	29.16	23.75	25.58	24.71	26.43	15.56	14.71	14.29	14.89

**Table 2:** Small sample of size $n = 500$ simulations (10$^3$ replications) from the bi-factor and second-order copula models with Gumbel copulas and group estimated average biases, root mean square errors (RMSE), and standard deviations (SD), scaled by *n*, for the ML estimates under different pair-copulas from the bi-factor and second-order copula models.

	Bi-factor copula model							
	$\tau (\varvec{\theta }_g), g=1,\ldots ,4$				$\tau (\varvec{\delta }_g), g=1,\ldots ,4$			
	0.45	0.55	0.65	0.75	0.30	0.35	0.40	0.50
*n*bias								
BVN	1.63	1.81	1.97	1.48	6.35	7.47	7.78	7.37
Gumbel	0.37	0.35	0.28	0.36	0.83	1.01	1.65	3.72
s.Gumbel	12.16	11.06	8.60	5.62	4.49	4.20	2.91	3.30
$t_5$	3.18	4.14	4.51	4.54	6.80	7.73	7.80	5.95
*n*SE								
BVN	14.86	13.53	12.20	11.07	30.16	31.51	32.84	41.35
Gumbel	15.08	13.80	12.32	10.96	29.60	31.36	32.85	43.02
s.Gumbel	15.47	13.75	12.15	10.95	33.85	35.76	40.00	57.10
$t_5$	15.19	13.70	12.13	10.77	31.04	32.03	32.90	40.13
*n*RMSE								
BVN	14.95	13.65	12.36	11.17	30.82	32.40	33.78	42.01
Gumbel	15.09	13.81	12.33	10.97	29.62	31.38	32.90	43.19
s.Gumbel	19.68	17.64	14.89	12.31	34.15	36.02	40.13	57.20
$t_5$	15.53	14.31	12.94	11.69	31.78	32.96	33.84	40.57

	Second-order copula model							
	$\tau (\varvec{\delta })$				$\tau (\varvec{\theta }_g), g=1,\ldots ,4$			
	0.30	0.35	0.40	0.45	0.40	0.50	0.60	0.70
*nbias*								
BVN	6.05	5.84	5.86	6.35	0.32	0.65	0.46	0.55
Gumbel	0.03	0.15	0.26	2.02	0.25	0.66	1.11	2.20
s.Gumbel	27.15	27.05	27.08	27.02	12.33	12.24	11.14	8.77
$t_5$	8.36	8.80	9.30	9.89	3.29	3.62	4.57	3.60
*nSE*								
BVN	22.88	25.09	25.09	27.34	16.81	16.43	17.01	20.99
Gumbel	22.50	24.71	25.44	27.70	16.92	16.73	17.54	21.52
s.Gumbel	25.77	27.19	27.12	29.78	17.63	16.55	16.47	18.18
$t_5$	23.45	25.55	25.64	27.75	17.28	16.68	17.25	20.70
*nRMSE*								
BVN	23.67	25.76	25.77	28.07	16.81	16.45	17.02	21.00
Gumbel	22.50	24.71	25.44	27.77	16.93	16.75	17.58	21.64
s.Gumbel	37.44	38.35	38.33	40.21	21.53	20.63	19.91	20.20
$t_5$	24.89	27.02	27.27	29.46	17.59	17.08	17.86	21.02

We randomly generate 1000 datasets with samples of size $n=500$ or 1000 and $d=16$ items, with $K=3$ or $K=5$ equally weighted categories, that are equally separated into $G=4$ non-overlapping groups from the bi-factor and second-order copula model. In each simulated model, we use different linking copulas to cover different types of dependence. To make the models comparable, we convert the BVN/$t_\nu$ and Gumbel/s.Gumbel copula parameters to Kendall’s $\tau$’s via the relations in (5) and (6), respectively. For the bi-factor copula models, we set $\tau (\varvec{\theta }_g)=(0.45,0.55,0.65,0.75)$ and $\tau (\varvec{\delta }_g)=(0.30,0.35,$ 0.40, 0.50) for $g=1,\ldots ,4$. For the second-order copula models, we set $\tau (\varvec{\theta }_g)=(0.4,0.5,0.6,0.7)$ for $g=1,\ldots ,4$ and $\tau (\varvec{\delta })=(0.30,0.35,0.40,0.45)$.

The Kendall’s tau parameters $\tau (\varvec{\theta }_g)$ and $\tau (\varvec{\delta }_g)$ as described above are common for each group; hence, Table 1 (Table 2) contains the group estimated average biases, root mean square errors (RMSE), and standard deviations (SD), scaled by *n*, for the IFM (ML) estimates under different pair-copulas from the bi-factor and second-order copula models. In the true (simulated) models, the linking copulas are Gumbel copulas. Given the large number of cutpoints as the number of categories *K* increases, for ML estimation we restrict ourselves to $K=3$ categories.

Conclusions from the values in the tables are the following:

**Table 3:** Small sample of size $n = 500$ simulations ($10^3$ replications) from the bi-factor and second-order copula models with various linking copulas and frequencies of the true bivariate copula identified using the model selection algorithm.

Bi-factor	Model 1			Model 2			Model 3			Model 4		
	Copula	$K=3$	$K=5$	Copula	$K=3$	$K=5$	Copula	$K=3$	$K=5$	Copula	$K=3$	$K=5$
$V_0$	Gumbel	992	1000	$t_5$	984	1000	Gumbel	996	1000	$t_5$	975	1000
$$ V_1$$	Gumbel	858	956	$$t_5$$	597	806	$$t_5$$	585	789	Gumbel	888	958
$$ V_2$$	Gumbel	870	951	$$t_5$$	588	799	$$t_5$$	569	775	Gumbel	894	969
$$ V_3$$	Gumbel	846	950	$$t_5$$	546	777	s.Gumbel	844	945	s.Gumbel	865	947
$$ V_4$$	Gumbel	844	942	$$t_5$$	589	805	s.Gumbel	878	949	s.Gumbel	900	956

Second-order	Model 1			Model 2			Model 3			Model 4		
	Copula	$K=3$	$K=5$	Copula	$K=3$	$K=5$	Copula	$K=3$	$K=5$	Copula	$K=3$	$K=5$
$V_0$	Gumbel	901	848	$t_5$	664	819	Gumbel	892	987	$t_5$	648	765
$V_1$	Gumbel	895	975	$t_5$	735	939	$t_5$	756	933	Gumbel	918	990
$V_2$	Gumbel	892	962	$t_5$	686	911	$t_5$	705	910	Gumbel	918	991
$V_3$	Gumbel	891	981	$t_5$	711	915	s.Gumbel	901	980	s.Gumbel	902	982
$V_4$	Gumbel	900	984	$t_5$	743	926	s.Gumbel	904	984	s.Gumbel	919	980

- IFM with the true bi-factor or second-order model is highly efficient according to the simulated biases, SDs and RMSEs.
- The IFM estimates of $\tau$’s are not robust under bivariate copula misspecification and their biases increase when the assumed bivariate copula has tail dependence of opposite direction from the true bivariate copula. For example, in Table 1 the scaled biases for the IFM estimates increase substantially when the linking copulas are the s.Gumbel copulas.
- IFM is not as punishing for bivariate copula misspecification as ML estimation. For example, the scaled biases for the ML estimates are even larger when the bivariate linking copulas are misspecified to the s.Gumbel copulas.

To examine the reliability of using the heuristic algorithm to select the true (simulated) bivariate linking copulas, samples of size 500 were generated from various bi-factor and second-order copula models. Table 3 presents the number of times that the true (simulated) bivariate linking copulas were chosen over 1000 simulation runs. It is revealed that the model selection algorithm performs extremely well for various bi-factor and second-order copulas models with different choices of linking copulas as the number of categories *K* increases. For a small *K* dependence in the tails cannot be easily quantified. Hence, for example, when the true copula is the $t_5$ which has the same upper and lower tail dependence, the algorithm selected either $t_5$ or BVN which has zero lower and upper tail dependence, because both copulas provide reflection symmetric dependence.

**Table 4:** Small sample of size $n =\{500,1000\}$ simulations (10$^3$ replications) from bi-factor and second-order copula models and the empirical rejection levels at $\alpha = \{0.20, 0.10, 0.05, 0.01\}$, degrees of freedom (df), mean and variance.

Bi-factor copula model			$M_2$						
Copula	*n*	K	df	Mean	Variance	$\alpha$=0.20	$\alpha$=0.10	$\alpha$=0.05	$\alpha$=0.01
BVN	500	3	448	449.0	912.8	0.206	0.100	0.060	0.016
		5	1888	1885.5	4858.3	0.210	0.117	0.065	0.024
	1000	3	448	448.7	879.0	0.192	0.097	0.051	0.020
		5	1888	1886.5	4332.5	0.202	0.108	0.064	0.015
Gumbel	500	3	448	449.9	887.3	0.216	0.111	0.053	0.011
		5	1888	1886.6	4709.7	0.225	0.126	0.070	0.015
	1000	3	448	448.9	864.0	0.201	0.102	0.050	0.015
		5	1888	1888.6	4332.1	0.226	0.107	0.069	0.014
$t_5$	500	3	448	448.7	907.3	0.202	0.088	0.048	0.018
		5	1888	1886.6	4479.4	0.204	0.107	0.053	0.017
	1000	3	448	448.6	834.9	0.184	0.090	0.050	0.014
		5	1888	1890.3	4008.5	0.218	0.103	0.052	0.015

Second copula model			$M_2$						
Copula	*n*	K	df	Mean	Variance	$\alpha$=0.20	$\alpha$=0.10	$\alpha$=0.05	$\alpha$=0.01
BVN	500	3	460	462.2	1001.2	0.220	0.113	0.055	0.016
		5	1900	1903.5	3736.2	0.214	0.112	0.052	0.010
	1000	3	460	461.3	1023.9	0.220	0.109	0.064	0.013
		5	1900	1906.5	3918.2	0.230	0.130	0.068	0.012
Gumbel	500	3	460	464.5	1011.3	0.233	0.117	0.073	0.024
		5	1900	1909.2	5099.8	0.245	0.129	0.064	0.008
	1000	3	460	461.9	871.2	0.203	0.106	0.049	0.009
		5	1900	1908.5	3977.0	0.239	0.129	0.067	0.015
$t_5$	500	3	460	465.3	1362.4	0.247	0.145	0.091	0.039
		5	1900	1904.7	3740.6	0.226	0.113	0.050	0.010
	1000	3	460	461.8	900.1	0.214	0.108	0.055	0.010
		5	1900	1908.1	3864.9	0.229	0.131	0.072	0.015

To check whether the $\chi ^2_{s-q}$ is a good approximation for the distribution of the $M_2$ statistic under the null hypothesis, samples of sizes 500 and 1000 were generated from various bi-factor second-order copula models. Table 4 contains four common nominal levels of the $M_2$ statistic under the bi-factor and second-order copula models with different bivariate copulas. As can be seen in the table, the observed levels of $M_2$ are close to the nominal $\alpha$ levels and remain accurate even for extremely sparse tables ($d=16$ and $K=5$).

## Application

The Toronto Alexithymia Scale (TAS) is the most utilized measure of alexithymia in empirical research (e.g., Bagby et al. 1994; Taylor et al. 2003; Parker et al. 2003; Gignac et al. 2007; Reise et al. 2013; Tuliao et al. 2020; Carnovale et al. 2021) and is composed of $d=20$ items. The aforementioned works suggest that the items measure 3 facets of alexithymia, namely Difficulty Identifying Feelings (DIF; $d_1=7$ items), Difficulty Describing Feelings (DDF; $d_2=5$ items), Externally Oriented Thinking (EOT; $d_3=8$ items), where each facet represents different non-overlapping items. Therefore, a 3-factor model was initially called, but given the hypothesized association among the three facets of the alexithymia construct, oblique (correlated) factor or bi-factor models have been used (e.g., Bagby et al. 1994; Parker et al. 2003; Gignac et al. 2007). Tuliao et al. (2020) has demonstrated that a bi-factor model outperforms any other competing factor model for the TAS scale. To this end, recent studies adopted the bi-factor structure for the TAS scale (e.g., Carnovale et al. 2021) and further supported a general alexithymia factor and group-specific factors (DIF, DDf and EOT) that account for homogeneous dependence amongst the non-overlapping items. Note also in passing that estimating a 3-factor or an oblique 3-factor model is computationally demanding as it requires 3-dimensional integration. This is not case for the bi-factor or second-order (an oblique 3-factor model where the group-specific factors are linked to another latent variable via a 1-factor model) models. In spite of the fact they involve $G+1=4$ latent variables, only require one-dimensional integrals of a function which in turn is a product of 3 one-dimensional integrals.

We use a dataset of 1925 university students from the French-speaking region of Belgium (Briganti and Linkowski 2020). Students were 17 to 25 years old, and 58% of them were female and 42% were male. They were asked to respond to each item using one of $K=5$ categories: “$1=$ completely disagree”, “$2=$ disagree”, “$3=$ neutral, “$4=$ agree”, “$5=$ completely agree”. The dataset and full description of the items can be found in the R package **BGGM** (Williams and Mulder 2020).

For these items, a respondent might be thinking about the average “sensation” of many past relevant events, leading to latent means. That is, based on the item descriptions, this seems more natural than a discretized maxima or minima. Since the sample is a mixture (male and female students), we can expect a priori that a bi-factor or second-order copula model with $t_\nu$ copulas might be plausible, as in this case the items can be considered as mixtures of discretized means.

**Table 5:** Average observed polychoric correlations and semi-correlations for all pairs within each group and for all pairs of items for the Toronto Alexithymia Scale (TAS), along with the corresponding theoretical semi-correlations for BVN, $t_5$, Frank, Gumbel , and survival Gumbel (s.Gumbel) copulas.

	All items			Items in group 1			Items in group 2			Items in group 3		
	$\rho _N$	$\rho _N^{-}$	$\rho _N^{+}$	$\rho _N$	$\rho _N^{-}$	$\rho _N^{+}$	$\rho _N$	$\rho _N^{-}$	$\rho _N^{+}$	$\rho _N$	$\rho _N^{-}$	$\rho _N^{+}$
Observed	0.17	0.21	0.20	0.34	0.36	0.29	0.42	0.37	0.40	0.19	0.26	0.29
BVN	0.17	0.07	0.07	0.34	0.16	0.16	0.42	0.21	0.21	0.19	0.08	0.08
$t_5$	0.17	0.23	0.23	0.34	0.31	0.31	0.42	0.35	0.35	0.19	0.24	0.24
Gumbel	0.17	0.05	0.22	0.34	0.11	0.37	0.42	0.14	0.43	0.19	0.05	0.24
s.Gumbel	0.17	0.22	0.05	0.34	0.37	0.11	0.42	0.43	0.14	0.19	0.24	0.05

In Table 5, we summarize the averages of polychoric semi-correlations for all pairs within each facet of alexithymia and for all pairs of items along with the theoretical semi-correlations in (8) under different choices of copulas. For a BVN/$t_\nu$ copula, the copula parameter is the sample polychoric correlation, while for a Gumbel/s.Gumbel copula the polychoric correlation was converted to Kendall’s tau with the relation in (5) and then from Kendall’s $\tau$ to Gumbel/s.Gumbel copula parameter via the functional inverse in (6). The summary of findings from the diagnostics in the table show that the items appear to be a mixed selection between discretized means and minima. For the indicators of the DIF factor (items in group 1) there is more correlation in the joint lower tail, i.e., the items are based on discretizations of latent variables that are minima and have more probability in the joint lower tail, suggesting the use of s.Gumbel linking copulas when modelling the responses to these items. All the other items have stronger correlation in both the joint upper and joint lower tail than with the BVN, i.e., the items are based on discretizations of latent variables that are means and have more probability in both the joint lower and upper tail, suggesting the use of $t_\nu$ bivariate linking copulas as the respondents consist of a “mixture” population (different genders), Hence, a bi-factor or second-order copula model with the aforementioned linking copulas might provide a better fit than the (Gaussian) models with BVN copulas.

Then, we fit the bi-factor and second-order models with the bivariate copulas selected by the heuristic algorithm in Sect. 3.2. For a baseline comparison, we also fit their special cases; these are the one- and two-factor copula models where we have also selected the bivariate copulas using the heuristic algorithm proposed by Kadhem and Nikoloulopoulos (2021). To show the improvement of the copula models over their Gaussian analogues, we have also fitted all the classes of copula models with BVN copulas. The fitted models are compared via the AIC, since the number of parameters is not the same between the models. In addition, we use Vuong’s test (Vuong 1989) to show if (a) the best fitted model according to the AICs provides better fit than the other fitted models and (b) a model with the selected copulas provides better fit than the one with BVN copulas. The Vuong’s test is the sample version of the difference in Kullback–Leibler divergence between two models and can be used to differentiate two parametric models which could be non-nested. For the Vuong’s test we provide the 95% confidence interval of the test statistic (Joe 2014, p. 258). If the interval does not contain 0, then the best fitted model according to the AICs is better if the interval is completely above 0. To assess the overall goodness-of-fit of the bi-factor and second-order copula models, we use the $M_2$ statistic, along with the Root Mean Square Error of Approximation based on $M_2$ (Maydeu-Olivares and Joe 2014), viz.

$$\begin{aligned} \text{ RMSEA}_2= \sqrt{\text{ Max } \left( \frac{{M}_2 - df}{n \times df},0\right) }. \end{aligned}$$

**Table 6:** AICs, Vuong’s 95% CIs, and $M_2$ statistics for the 1-factor, 2-factor, bi-factor and second-order copula models with BVN copulas and selected copulas, along with the maximum deviations of observed and expected counts for all pairs within each group and for all pairs of items for the Toronto Alexithymia Scale.

	1-factor		2-factor		Bi-factor		Second-order	
	BVN	Selected	BVN	Selected	BVN	Selected	BVN	Selected
AIC	107135.8	105504.0	106189.5	103893.5	105507.7	103200.9	105878.6	104133.7
Vuong’s 95% $\hbox {CI}^a$	(0.35,0.50)		(0.53,0.69)		(0.51,0.69)		(0.38,0.52)	
Vuong’s 95% $\hbox {CI}^b$	(0.93,1.13)	(0.55,0.67)	(0.69,0.88)	(0.13,0.23)	(0.51,0.69)		(0.61,0.80)	(0.21,0.29)
$M_2$	14723.8	9865.0	9195.7	7383.7	11664.7	6381.5	13547.1	7341.2
df	3020	3020	3001	3000	3000	3000	3017	3017
RMSEA$_2$	0.045	0.034	0.033	0.028	0.039	0.024	0.043	0.027
Maximum discrepancy								
Items in Group 1	71	63	71	60	69	55	70	61
Items in Group 2	112	98	113	83	77	48	84	55
Items in Group 3	87	74	81	52	80	45	82	53
All items	112	98	113	83	80	55	84	61

$^\textrm{a}\hbox {Selected}$ factor copula model versus its Gaussian special case.

$^\textrm{b}\hbox {Selected}$ Bi-factor copula model versus any other fitted model.

Table 6 gives the AICs, the 95% CIs of Vuong’s tests and the $M_2$ statistics for all the fitted models. The best fitted model, based on AIC values, is the bi-factor copula model obtained from the selection algorithm. The best-fitted bi-factor copula model results when we use s.Gumbel for the DIF factor, $t_3$ for both the DDF and EOT factors and $t_2$ for the common factor (alexithymia). This is in line with the preliminary analyses based on the interpretations of items as mixtures of means and the diagnostics in Table 5. The proposed model selection algorithm has selected the $t_\nu$ copula that has the same lower and upper tail dependence for the common and all the group specific factors except the group specific factor in group 1 for which the survival Gumbel copula has been selected. For the items in group 1, the largest difference ${\hat{\rho }}_N^--{\hat{\rho }}_N^+=0.07$ is found showing more dependence in the lower tail and the fact that for this group the survival Gumbel copula is selected it shows that this difference is statistically significant. No other difference is statistically significant. It is revealed that the DIF items and DIF factor are discretized and latent minima, respectively, as the participants seem to reflect that they “disagree” or “completely disagree” having difficulty identifying feelings. From the Vuong’s 95% Cls and $M_2$ statistics, it is shown that factor copula models provide a big improvement over their Gaussian analogues and that the selected bi-factor copula model outperforms all the fitted models.

**Table 7:** Estimated copula parameters and their standard errors (SE) in Kendall’s $\tau$ scale for the bi-factor copula models with BVN copulas and selected copulas for the Toronto Alexithymia Scale.

Items	Bi-factor copula model with BVN copulas				Bi-factor copula model with selected copulas					
	Common factor		Group-specific factors		Common factor			Group-specific factors		
	Est	SE	Est	SE	Copulas	Est	SE	Copulas	Est	SE
1	0.42	0.01	0.23	0.02	$t_2$	0.49	0.02	s.Gumbel	0.09	0.03
3	0.14	0.02	0.24	0.02	$t_2$	0.16	0.02	s.Gumbel	0.37	0.02
6	0.22	0.02	0.29	0.02	$t_2$	0.29	0.02	s.Gumbel	0.23	0.02
7	0.11	0.02	0.31	0.02	$t_2$	0.09	0.02	s.Gumbel	0.53	0.04
9	0.38	0.01	0.34	0.02	$t_2$	0.47	0.02	s.Gumbel	0.24	0.02
13	0.36	0.01	0.46	0.02	$t_2$	0.49	0.02	s.Gumbel	0.32	0.03
14	0.21	0.02	0.36	0.02	$t_2$	0.30	0.02	s.Gumbel	0.27	0.03
2	0.71	0.02	− 0.24	0.10	$t_2$	0.46	0.02	$t_3$	0.53	0.02
4	0.55	0.01	0.02	0.04	$t_2$	0.41	0.02	$t_3$	0.58	0.03
11	0.35	0.01	0.13	0.03	$t_2$	0.33	0.02	$t_3$	0.20	0.03
12	0.34	0.02	0.29	0.04	$t_2$	0.29	0.02	$t_3$	0.23	0.03
17	0.31	0.02	0.38	0.06	$t_2$	0.24	0.02	$t_3$	0.25	0.03
5	0.06	0.02	0.33	0.02	$t_2$	0.10	0.02	$t_3$	0.34	0.02
8	0.11	0.02	0.30	0.02	$t_2$	0.16	0.02	$t_3$	0.33	0.02
10	0.12	0.02	0.27	0.02	$t_2$	0.14	0.02	$t_3$	0.30	0.02
15	0.15	0.02	0.19	0.02	$t_2$	0.12	0.02	$t_3$	0.19	0.02
16	0.03	0.02	0.23	0.02	$t_2$	0.03	0.02	$t_3$	0.24	0.02
18	− 0.02	0.02	0.28	0.02	$t_2$	0.03	0.02	$t_3$	0.29	0.02
19	0.07	0.02	0.40	0.02	$t_2$	0.10	0.02	$t_3$	0.43	0.02
20	0.06	0.02	0.27	0.02	$t_2$	0.10	0.02	$t_3$	0.26	0.02

The highly statistical significant $M_2$ statistics are not surprising since one should expect discrepancies between the postulated parametric model and the population probabilities, when the sample size or dimension is sufficiently large (Maydeu-Olivares and Joe 2014) as in our case; none should expect a model with 3000 df to fit exactly. To further show that the fit has been improved we have calculated the maximum deviations of observed and model-based counts for each bivariate margin, that is, $D_{j_1j_2}=n\max _{y_1,y_2}|p_{j_1,j_2,y_1,y_2}-\pi _{j_1,j_2,y_1,y_2}({\hat{\varvec{\theta }}})|$. In Table 6, we summarize the averages of these deviations for all pairs within each group and for all pairs of items. Overall, the maximum discrepancies have been sufficiently reduced in the selected bi-factor model.

Table 7 gives the copula parameter estimates in Kendall’s $\tau$ scale and their standard errors (SE) for the selected bi-factor copula model and the Gaussian bi-factor model as the benchmark model. The SEs of the estimated parameters are obtained by the inversion of the Hessian matrix at the second step of the IFM method. These SEs are adequate to assess the flatness of the log-likelihood. Proper SEs that account for the estimation of cutpoints can be obtained by jackknifing the two-stage estimation procedure. The loading parameters (${\hat{\tau }}$’s converted to BVN copula parameters via the functional inverse in (5) and then to loadings using the relations in Section 2.3) show that the common alexithymia factor is mostly loaded on DIF and DDF items, suggesting that items in the domains DIF and DDF are good indicators for alexithymia. The items in the EOT although they loaded on the EOT latent factor, they had poor loadings in the common alexithymia factor.

## Discussion

For items from several domains, we have proposed bi-factor and second-order copula models where we replace BVN distributions, between observed and latent variables, with bivariate copulas. Our copula constructions include the Gaussian bi-factor and second-order models as special cases and can provide a substantial improvement over the Gaussian models based on AIC, Vuong’s and goodness-of-fit statistics. Hence, superior statistical inference for the loadings can be achieved. We have demonstrated that the Kendall’s $\tau$’s or loading parameters are not robust under bivariate linking copula misspecification and their biases increase when the assumed bivariate copula has tail dependence of opposite direction from the true bivariate copula.

The improvement relies on the fact that when we use appropriate bivariate copulas other than BVN copulas in the construction, there is an interpretation of latent variables that can be maxima/minima or mixture of means instead of means. The bi-factor and second-order copula models, if other than BVN copulas are called, have a latent structure that is not additive as in (3) and (4), respectively. The bi-factor copula (dependence) parameters are interpretable as dependence of an observed variable with the common factor, or conditional dependence of an observed variable with the group-specific latent variable given the common factor, i.e., the bi-factor copula model permits conditional dependence within identified subsets of items.

Both the bi-factor and second-order copula models lead to substantial simplification of the joint likelihood. The joint pmfs in (1) and (2) reduce to one-dimensional integrals of a function which in turn is a product of *G* one-dimensional integrals. Hence, the evaluation of the joint likelihood requires only low-dimensional integration, as in the one- and two-factor copula models, regardless of the dimension $G+1$ of the factors. This is an advantage over the *p*-factor ($p>2$) copula models where the joint pmf requires *p*-dimensional integration and becomes intractable as the number of factors increases. Hence, the proposed structured multidimensional factor models provide parsimonious factor solutions without any computational deficiencies as in the *p*-factor copula models when *p* increases.

We have proposed a numerically stable likelihood estimation technique based on Gauss–Legendre quadrature. The use of independent Gauss–Legendre quadrature points for this kind of models has been proposed in Krupskii and Joe (2015). For the bi-factor copula models for item response the integrants are bounded and thus independent Gauss–Legendre points are fine. For the second-order copula models for item response the integrants can be unbounded as copula densities can be unbounded, hence we have proposed the novel use of dependent Gauss–Legendre quadrature points that have an 1-factor copula distribution.

Building on the models proposed in this paper, there are several extensions that can be implemented. The adoption of the structure of the Gaussian tri-factor and third-order models (e.g., Rijmen et al. 2014), to account for any additional layer of dependence, is feasible using the notion of truncated vine copulas that involve both observed and latent variables.

## Software

R functions for estimation, simulation, model selection and goodness-of-fit of the bi-factor and second-order copula models are part of the R package **FactorCopula** (Kadhem and Nikoloulopoulos 2022). All the analyses presented in Sect. 6 are given as code examples in the package.

## Supplementary Information

Below is the link to the electronic supplementary material.

Supplementary Information: We provide the form of the derivatives of the univariate and bivariate marginal probabilities with respect to the estimated model parameters in the Supplementary Tables 1--5 of the electronic supplementary material. (pdf 66 KB)
