# Supplementary material for: Bi-factor and Second-Order Copula Models for Item Response Data
Source: Psychometrika. 2022 Nov 21;88(1):132–57. doi: 10.1007/s11336-022-09894-2 (PMC9977904; doi:10.1007/s11336-022-09894-2)
Supplement: Supplementary file 1 — Supplementary Information: We provide the form of the derivatives of the univariate and bivariate marginal probabilities with respect to the estimated model parameters in the Supplementary Tables 1--5 of the electronic supplementary material. (pdf 66 KB) [file 11336_2022_9894_MOESM1_ESM.pdf]

Supplementary Information for

Bi-factor and second-order copula models for item response data

by

Sayed H. Kadhem and Aristidis K. Nikoloulopoulos*

*a.nikoloulopoulos@uea.ac.uk, School of Computing Sciences, University of East Anglia, Norwich NR4 7TJ, U.K.

Supplementary Table 1: Derivatives of the univariate probability $\pi_{jg,y} = \Phi(\alpha_{jg,y+1}) - \Phi(\alpha_{jg,y})$ with respect to the cutpoint $\alpha_{jg,k}$ for $g = 1\ldots, G,\ j = 1, \ldots, d_g,\ y = 1, \ldots, K-1,$ and $k = 1, \ldots, K-1$.

$\partial\pi_{jg,y}/\partial\alpha_{jg,k}$	If
$\phi(\alpha_{jg,y+1})$	$k = y + 1$
$-\phi(\alpha_{jg,y})$	$k = y$

Supplementary Table 2: Derivatives of the bivariate probability $\pi_{j_1j_2g,y_1,y_2} = \Pr(Y_{j_1g} = y_1, Y_{j_2g} = y_2)$ with respect to the cutpoint $\alpha_{jg,k}$, the copula parameter θ_{jg} for the common factor V_0, and the copula parameter δ_{jg} for the group-specific factor V_g for the bi-factor copula model for $g = 1\ldots,G$, $j, j_1, j_2 = 1,\ldots,d_g$, $y, y_1, y_2 = 1,\ldots,K-1$, and $k = 1,\ldots,K-1$. Note that $f_{Y_{jg}|V_g;V_0}(y_{jg}|v_g;v_0) = \Big(C_{Y_{jg}|V_g;V_0}\big(C_{Y_{jg}|V_0}(a_{jg,y+1}|v_0;\theta_{jg})|v_g;\delta_{jg}\big) - C_{Y_{jg}|V_g;V_0}\big(C_{Y_{jg}|V_0}(a_{jg,y}|v_0;\theta_{jg})|v_g;\delta_{jg}\big)\Big)$ where $a_{jg,k} = \Phi(\alpha_{jg,k})$, $c_{V_0Y_{jg}}(v_0,a) = \partial^2 C_{V_0Y_{jg}}(v_0,a)/\partial v_0\partial a$, $\dot{C}_{jg|V_0}(\cdot;\theta_{jg}) = \partial C_{jg|V_0}(\cdot;\theta_{jg})/\partial\theta_{jg}$, $\dot{C}_{Y_{jg}|V_g;V_0}(\cdot;\delta_{jg}) = \partial C_{Y_{jg}|V_g;V_0}(\cdot;\delta_{jg})/\partial\delta_{jg}$, $\dot{f}_{Y_{jg}|V_{jg};V_0}(y_{jg}|v_g;v_0) = \partial f_{Y_{jg}|V_{jg};V_0}(y_{jg}|v_g;v_0)/\partial\delta_{jg} = \dot{C}_{Y_{jg}|V_g;V_0}\big(C_{Y_{jg}|V_0}(a_{jg,y+1}|v_0)|v_g\big) - \dot{C}_{Y_{jg}|V_g;V_0}\big(C_{Y_{jg}|V_0}(a_{jg,y}|v_0)|v_g\big)$, $\bar{f}_{Y_{jg}|V_{jg};V_0}(y_{jg}|v_g;v_0) = \partial f_{Y_{jg}|V_{jg};V_0}(y_{jg}|v_g;v_0)/\partial\theta_{jg} = c_{V_gY_{jg}}\big(v_g, C_{Y_{jg}|V_0}(a_{jg,y+1}|v_0)\big)\dot{C}_{Y_{jg}|V_0}(a_{jg,y+1}|v_0) - c_{V_gY_{jg}}\big(v_g, C_{Y_{jg}|V_0}(a_{jg,y}|v_0)\big)\dot{C}_{Y_{jg}|V_0}(a_{jg,y}|v_0)$.

$\partial\pi_{j_1j_2g,y_1,y_2}/\partial\alpha_{jg,k}$	If
$\phi(\alpha_{j_1g,y_1+1})\int_0^1\int_0^1 f_{Y_{j_2g}\|V_g;V_0}(y_{j_2g}\|v_g;v_0)\ c_{V_gY_{j_1g}}\big(v_g, C_{Y_{j_1g}\|V_0}(a_{j_1g,y_1+1}\|v_0)\big)\ c_{V_0Y_{j_1g}}(v_0, a_{j_1g,y_1+1})\ dv_g dv_0$	$j = j_1, k = y_1+1$
$-\phi(\alpha_{j_1g,y_1})\int_0^1\int_0^1 f_{Y_{j_2g}\|V_g;V_0}(y_{j_2g}\|v_g;v_0)\ c_{V_gY_{j_1g}}\big(v_g, C_{Y_{j_1g}\|V_0}(a_{j_1g,y_1}\|v_0)\big)\ c_{V_0Y_{j_1g}}(v_0, a_{j_1g,y_1})\ dv_g dv_0$	$j = j_1, k = y_1$
$\phi(\alpha_{j_2g,y_2+1})\int_0^1\int_0^1 f_{Y_{j_1g}\|V_g;V_0}(y_{j_1g}\|v_g;v_0)\ c_{V_gY_{j_2g}}\big(v_g, C_{Y_{j_2g}\|V_0}(a_{j_2g,y_2+1}\|v_0)\big)\ c_{V_0Y_{j_2g}}(v_0, a_{j_2g,y_2+1})\ dv_g dv_0$	$j = j_2, k = y_2+1$
$-\phi(\alpha_{j_2g,y_2})\int_0^1\int_0^1 f_{Y_{j_1g}\|V_g;V_0}(y_{j_1g}\|v_g;v_0)\ c_{V_gY_{j_2g}}\big(v_g, C_{Y_{j_2g}\|V_0}(a_{j_2g,y_2}\|v_0)\big)\ c_{V_0Y_{j_2g}}(v_0, a_{j_2g,y_2})\ dv_g dv_0$	$j = j_2, k = y_2$
$\partial\pi_{j_1j_2g,y_1,y_2}/\partial\theta_{jg}$	If
$\int_0^1\int_0^1 f_{Y_{j_2g}\|V_g;V_0}(y_{j_2g}\|v_g;v_0)\ \bar{f}_{Y_{j_1g}\|V_{j_1g};V_0}(y_{j_1g}\|v_g;v_0)\ dv_g dv_0$	$j = j_1$
$\int_0^1\int_0^1 f_{Y_{j_1g}\|V_g;V_0}(y_{j_1g}\|v_g;v_0)\ \bar{f}_{Y_{j_2g}\|V_{j_2g};V_0}(y_{j_2g}\|v_g;v_0)\ dv_g dv_0$	$j = j_2$
$\partial\pi_{j_1j_2g,y_1,y_2}/\partial\delta_{jg}$	If
$\int_0^1\int_0^1 f_{Y_{j_2g}\|V_g;V_0}(y_{j_2g}\|v_g;v_0)\ \dot{f}_{Y_{j_1g}\|V_{j_1g};V_0}(y_{j_1g}\|v_g;v_0)\ dv_g dv_0$	$j = j_1$
$\int_0^1\int_0^1 f_{Y_{j_1g}\|V_g;V_0}(y_{j_1g}\|v_g;v_0)\ \dot{f}_{Y_{j_2g}\|V_{j_2g};V_0}(y_{j_2g}\|v_g;v_0)\ dv_g dv_0$	$j = j_2$

Supplementary Table 3: Derivatives of the bivariate probability $\pi_{j_1g_1j_2g_2,y_1,y_2} = \Pr(Y_{j_1g_1} = y_1, Y_{j_2g_2} = y_2)$ with respect to the cutpoint $\alpha_{jg,k}$, the copula parameter θ_{jg} for the common factor V_0, and the copula parameter δ_{jg} for the group-specific factor V_g for the bi-factor copula model for $g = 1\ldots,G$, $j,j_1,j_2 = 1,\ldots,d_g$, $y,y_1,y_2 = 1,\ldots,K-1$, and $k = 1,\ldots,K-1$. Note that $f_{Y_{jg}|V_g;V_0}(y_{jg}|v_g;v_0) = \Big(C_{Y_{jg}|V_g;V_0}\big(C_{Y_{jg}|V_0}(a_{jg,y+1}|v_0;\theta_{jg})|v_g;\delta_{jg}\big) - C_{Y_{jg}|V_g;V_0}\big(C_{Y_{jg}|V_0}(a_{jg,y}|v_0;\theta_{jg})|v_g;\delta_{jg}\big)\Big)$ where $a_{jg,k} = \Phi(\alpha_{jg,k})$, $c_{V_0Y_{jg}}(v_0,a) = \partial^2 C_{V_0Y_{jg}}(v_0,a)/\partial v_0\partial a$, $\dot{C}_{jg|V_0}(\cdot;\theta_{jg}) = \partial C_{jg|V_0}(\cdot;\theta_{jg})/\partial\theta_{jg}$, $\dot{C}_{Y_{jg}|V_g;V_0}(\cdot;\delta_{jg}) = \partial C_{Y_{jg}|V_g;V_0}(\cdot;\delta_{jg})/\partial\delta_{jg}$, $\dot{f}_{Y_{jg}|V_{jg};V_0}(y_{jg}|v_g;v_0) = \partial f_{Y_{jg}|V_{jg};V_0}(y_{jg}|v_g;v_0)/\partial\delta_{jg} = \dot{C}_{Y_{jg}|V_g;V_0}\big(C_{Y_{jg}|V_0}(a_{jg,y+1}|v_0)|v_g\big) - \dot{C}_{Y_{jg}|V_g;V_0}\big(C_{Y_{jg}|V_0}(a_{jg,y}|v_0)|v_g\big)$, $\bar{f}_{Y_{jg}|V_{jg};V_0}(y_{jg}|v_g;v_0) = \partial f_{Y_{jg}|V_{jg};V_0}(y_{jg}|v_g;v_0)/\partial\theta_{jg} = c_{V_gY_{jg}}\big(v_g, C_{Y_{jg}|V_0}(a_{jg,y+1}|v_0)\big)\dot{C}_{Y_{jg}|V_0}(a_{jg,y+1}|v_0) - c_{V_gY_{jg}}\big(v_g, C_{Y_{jg}|V_0}(a_{jg,y}|v_0)\big)\dot{C}_{Y_{jg}|V_0}(a_{jg,y}|v_0)$.

$\partial\pi_{j_1g_1j_2g_2,y_1,y_2}/\partial\alpha_{jg,k}$	If
$\phi(\alpha_{j_1g_1,y_1+1})\int_0^1\int_0^1 f_{Y_{j_2g_2}|V_{g_2};V_0}(y_{j_2g_2}|v_{g_2};v_0)\ dv_{g_2}\int_0^1 c_{V_{g_1}Y_{j_1g_1}}\big(v_{g_1}, C_{Y_{j_1g_1}|V_0}(a_{j_1g_1,y_1+1}|v_0)\big)\ c_{V_0Y_{j_1g_1}}(v_0, a_{j_1g_1,y_1+1})\ dv_{g_1}dv_0$	$j=j_1, g=g_1, k=y_1+1$
$-\phi(\alpha_{j_1g_1,y_1})\int_0^1\int_0^1 f_{Y_{j_2g_2}|V_{g_2};V_0}(y_{j_2g_2}|v_{g_2};v_0)\ dv_{g_2}\int_0^1 c_{V_{g_1}Y_{j_1g_1}}\big(v_{g_1}, C_{Y_{j_1g_1}|V_0}(a_{j_1g_1,y_1}|v_0)\big)\ c_{V_0Y_{j_1g_1}}(v_0, a_{j_1g_1,y_1})\ dv_{g_1}dv_0$	$j=j_1, g=g_1, k=y_1$
$\phi(\alpha_{j_2g_2,y_2+1})\int_0^1\int_0^1 f_{Y_{j_1g_1}|V_{g_1};V_0}(y_{j_1g_1}|v_{g_1};v_0)\ dv_{g_1}\int_0^1 c_{V_{g_2}Y_{j_2g_2}}\big(v_{g_2}, C_{Y_{j_2g_2}|V_0}(a_{j_2g_2,y_2+1}|v_0)\big)\ c_{V_0Y_{j_2g_2}}(v_0, a_{j_2g_2,y_2+1})\ dv_{g_2}dv_0$	$j=j_2, g=g_2, k=y_2+1$
$-\phi(\alpha_{j_2g_2,y_2})\int_0^1\int_0^1 f_{Y_{j_1g_1}|V_{g_1};V_0}(y_{j_1g_1}|v_{g_1};v_0)\ dv_{g_1}\int_0^1 c_{V_{g_2}Y_{j_2g_2}}\big(v_{g_2}, C_{Y_{j_2g_2}|V_0}(a_{j_2g_2,y_2}|v_0)\big)\ c_{V_0Y_{j_2g_2}}(v_0, a_{j_2g_2,y_2})\ dv_{g_2}dv_0$	$j=j_2, g=g_2, k=y_2$
$\partial\pi_{j_1g_1j_2g_2,y_1,y_2}/\partial\theta_{jg}$	If
$\int_0^1\int_0^1 f_{Y_{j_2g_2}|V_{g_2};V_0}(y_{j_2g_2}|v_{g_2};v_0)\ dv_{g_2}\int_0^1 \bar{f}_{Y_{j_1g_1}|V_{j_1g_1};V_0}(y_{j_1g_1}|v_{g_1};v_0)\ dv_{g_1}dv_0$	$j=j_1, g=g_1$
$\int_0^1\int_0^1 f_{Y_{j_1g_1}|V_{g_1};V_0}(y_{j_1g_1}|v_{g_1};v_0)\ dv_{g_1}\int_0^1 \bar{f}_{Y_{j_2g_2}|V_{j_2g_2};V_0}(y_{j_2g_2}|v_{g_2};v_0)\ dv_{g_2}dv_0$	$j=j_2, g=g_2$
$\partial\pi_{j_1g_1j_2g_2,y_1,y_2}/\partial\delta_{jg}$	If
$\int_0^1\int_0^1 f_{Y_{j_2g_2}|V_{g_2};V_0}(y_{j_2g_2}|v_{g_2};v_0)\ dv_{g_2}\int_0^1 \dot{f}_{Y_{j_1g_1}|V_{j_1g_1};V_0}(y_{j_1g_1}|v_{g_1};v_0)\ dv_{g_1}dv_0$	$j=j_1, g=g_1$
$\int_0^1\int_0^1 f_{Y_{j_1g_1}|V_{g_1};V_0}(y_{j_1g_1}|v_{g_1};v_0)\ dv_{g_1}\int_0^1 \dot{f}_{Y_{j_2g_2}|V_{j_2g_2};V_0}(y_{j_2g_2}|v_{g_2};v_0)\ dv_{g_2}dv_0$	$j=j_2, g=g_2$

Supplementary Table 4: Derivatives of the bivariate probabilities $\pi_{j_1 j_2 g, y_1, y_2} = \Pr(Y_{j_1 g} = y_1, Y_{j_2 g} = y_2)$ with respect to the cutpoint $\alpha_{jg,k}$, the copula parameter θ_{jg} for the first-order factor V_g, and the copula parameter δ_g for the the second-order factor V_0 for the second-order copula model for $g = 1\ldots, G,\ \ j, j_1, j_2 = 1, \ldots, d_g,\ \ y, y_1, y_2 = 1, \ldots, K-1,$ and $k = 1, \ldots, K-1$. Note that $f_{Y_{jg}|V_g}(y_{jg}|v_g) = C_{Y_{jg}|V_g}\big(a_{jg,y+1}|v_g;\theta_{jg}\big) - C_{Y_{jg}|V_g}\big(a_{jg,y}|v_g;\theta_{jg}\big)$, $c_{V_g Y_{jg}}(v_g, a) = \partial^2 C_{V_g Y_{jg}}(v_g, a)/\partial v_g \partial a$, $\dot{C}_{Y_{jg}|V_g}(\cdot;\theta_{jg}) = \partial C_{Y_{jg}|V_g}(\cdot;\theta_{jg})/\partial\theta_{jg}$, $\dot{f}_{Y_{jg}|V_{jg}}(y_{jg}|v_g) = \partial f_{Y_{jg}|V_{jg}}(y_{jg}|v_g)/\partial\theta_{jg} = \dot{C}_{Y_{jg}|V_g}\big(a_{jg,y+1}|v_g\big) - \dot{C}_{Y_{jg}|V_g}\big(a_{jg,y}|v_g\big)$, $\dot{c}_{V_g V_0}(v_g, v_0;\delta_g) = \partial c_{V_g V_0}(v_g, v_0;\delta_g)/\partial\delta_g$.

$\partial\pi_{j_1 j_2 g, y_1, y_2}/\partial\alpha_{jg,k}$	If
$\phi(\alpha_{j_1 g, y_1+1}) \int_0^1 \int_0^1 f_{Y_{j_2 g}\|V_g}(y_{j_2 g}\|v_g)\ c_{V_g Y_{j_1 g}}(v_g, a_{j_1 g, y_1+1})\ c_{V_g V_0}(v_g, v_0)\ dv_g dv_0$	$j = j_1, k = y_1 + 1$
$-\phi(\alpha_{j_1 g, y_1}) \int_0^1 \int_0^1 f_{Y_{j_2 g}\|V_g}(y_{j_2 g}\|v_g)\ c_{V_g Y_{j_1 g}}(v_g, a_{j_1 g, y_1})\ c_{V_g V_0}(v_g, v_0)\ dv_g dv_0$	$j = j_1, k = y_1$
$\phi(\alpha_{j_2 g, y_2+1}) \int_0^1 \int_0^1 f_{Y_{j_1 g}\|V_g}(y_{j_1 g}\|v_g)\ c_{V_g Y_{j_2 g}}(v_g, a_{j_2 g, y_2+1})\ c_{V_g V_0}(v_g, v_0)\ dv_g dv_0$	$j = j_2, k = y_2 + 1$
$-\phi(\alpha_{j_2 g, y_2}) \int_0^1 \int_0^1 f_{Y_{j_1 g}\|V_g}(y_{j_1 g}\|v_g)\ c_{V_g Y_{j_2 g}}(v_g, a_{j_2 g, y_2})\ c_{V_g V_0}(v_g, v_0)\ dv_g dv_0$	$j = j_2, k = y_2$
$\partial\pi_{j_1 j_2 g, y_1, y_2}/\partial\theta_{jg}$	If
$\int_0^1 \int_0^1 f_{Y_{j_2 g}\|V_g}(y_{j_2 g}\|v_g)\ \dot{f}_{Y_{j_1 g}\|V_g}(y_{j_1 g}\|v_g)\ c_{V_g V_0}(v_g, v_0)\ dv_g dv_0$	$j = j_1$
$\int_0^1 \int_0^1 f_{Y_{j_1 g}\|V_g}(y_{j_1 g}\|v_g)\ \dot{f}_{Y_{j_2 g}\|V_g}(y_{j_2 g}\|v_g)\ c_{V_g V_0}(v_g, v_0)\ dv_g dv_0$	$j = j_2$
$\partial\pi_{j_1 j_2 g, y_1, y_2}/\partial\delta_g$	
$\int_0^1 \int_0^1 f_{Y_{j_1 g}\|V_g}(y_{j_1 g}\|v_g)\ f_{Y_{j_2 g}\|V_g}(y_{j_2 g}\|v_g)\ \dot{c}_{V_g V_0}(v_g, v_0)\ dv_g dv_0$	

Supplementary Table 5: Derivatives of the bivariate probability $\pi_{j_1g_1j_2g_2,y_1,y_2} = \Pr(Y_{j_1g_1} = y_1, Y_{j_2g_2} = y_2)$ *with respect to the cutpoint* $\alpha_{jg,k}$*, the copula parameter* θ_{jg} *for the first-order factor* V_g*, and the copula parameter* δ_g *for the second-order factor* V_0 *for the second-order copula model for* $g = 1\ldots,G,\ \ j,j_1,j_2 = 1,\ldots,d_g,\ \ y,y_1,y_2 = 1,\ldots,K-1,$ *and* $k = 1,\ldots,K-1$*. Note that* $f_{Y_{jg}|V_g}(y_{jg}|v_g) = C_{Y_{jg}|V_g}\big(a_{jg,y+1}|v_g;\theta_{jg}\big) - C_{Y_{jg}|V_g}\big(a_{jg,y}|v_g;\theta_{jg}\big),\ \ c_{V_gY_{jg}}(v_g,a) = \partial^2 C_{V_gY_{jg}}(v_g,a)/\partial v_g\partial a,\ \ \dot{C}_{Y_{jg}|V_g}(\cdot;\theta_{jg}) = \partial C_{Y_{jg}|V_g}(\cdot;\theta_{jg})/\partial\theta_{jg},\ \ \dot{f}_{Y_{jg}|V_{jg}}(y_{jg}|v_g) = \partial f_{Y_{jg}|V_{jg}}(y_{jg}|v_g)/\partial\theta_{jg} = \dot{C}_{Y_{jg}|V_g}\big(a_{jg,y+1}|v_g\big) - \dot{C}_{Y_{jg}|V_g}\big(a_{jg,y}|v_g\big),\ \ \dot{c}_{V_gV_0}(v_g,v_0;\delta_g) = \partial c_{V_gV_0}(v_g,v_0;\delta_g)/\partial\delta_g.$

$\partial\pi_{j_1g_1j_2g_2,y_1,y_2}/\partial\alpha_{jg,k}$	If
$\phi(\alpha_{j_1g_1,y_1+1})\int_0^1\int_0^1\ f_{Y_{j_2g_2}\|V_{g_2}}(y_{j_2g_2}\|v_{g_2})\ c_{V_{g_2}V_0}(v_{g_2},v_0)\ dv_{g_2}\int_0^1\ c_{V_{g_1}Y_{j_1g_1}}(v_{g_1},a_{j_1g_1,y_1+1})\ c_{V_{g_1}V_0}(v_{g_1},v_0)\ dv_{g_1}dv_0$	$j=j_1, g=g_1, k=y_1+1$
$-\phi(\alpha_{j_1g_1,y_1})\int_0^1\int_0^1\ f_{Y_{j_2g_2}\|V_{g_2}}(y_{j_2g_2}\|v_{g_2})\ c_{V_{g_2}V_0}(v_{g_2},v_0)\ dv_{g_2}\int_0^1\ c_{V_{g_1}Y_{j_1g_1}}(v_{g_1},a_{j_1g_1,y_1})\ c_{V_{g_1}V_0}(v_{g_1},v_0)\ dv_{g_1}dv_0$	$j=j_1, g=g_1, k=y_1$
$\phi(\alpha_{j_2g_2,y_2+1})\int_0^1\int_0^1\ f_{Y_{j_1g_1}\|V_{g_1}}(y_{j_1g_1}\|v_{g_1})\ c_{V_{g_1}V_0}(v_{g_1},v_0)\ dv_{g_1}\int_0^1\ c_{V_{g_2}Y_{j_2g_2}}(v_{g_2},a_{j_2g_2,y_2+1})\ c_{V_{g_2}V_0}(v_{g_2},v_0)\ dv_{g_2}dv_0$	$j=j_2, g=g_2, k=y_2+1$
$-\phi(\alpha_{j_2g_2,y_2})\int_0^1\int_0^1\ f_{Y_{j_1g_1}\|V_{g_1}}(y_{j_1g_1}\|v_{g_1})\ c_{V_{g_1}V_0}(v_{g_1},v_0)\ dv_{g_1}\int_0^1\ c_{V_{g_2}Y_{j_2g_2}}(v_{g_2},a_{j_2g_2,y_2})\ c_{V_{g_2}V_0}(v_{g_2},v_0)\ dv_{g_2}dv_0$	$j=j_2, g=g_2, k=y_2$
$\partial\pi_{j_1g_1j_2g_2,y_1,y_2}/\partial\theta_{jg}$	If
$\int_0^1\int_0^1\ f_{Y_{j_2g_2}\|V_{g_2}}(y_{j_2g_2}\|v_{g_2})\ c_{V_{g_2}V_0}(v_{g_2},v_0)\ dv_{g_2}\int_0^1\ \dot{f}_{Y_{j_1g_1}\|V_{g_1}}(y_{j_1g_1}\|v_{g_1})\ c_{V_{g_1}V_0}(v_{g_1},v_0)\ dv_{g_1}dv_0$	$j=j_1, g=g_1$
$\int_0^1\int_0^1\ f_{Y_{j_1g_1}\|V_{g_1}}(y_{j_1g_1}\|v_{g_1})\ c_{V_{g_1}V_0}(v_{g_1},v_0)\ dv_{g_1}\int_0^1\ \dot{f}_{Y_{j_2g_2}\|V_{g_2}}(y_{j_2g_2}\|v_{g_2})\ c_{V_{g_2}V_0}(v_{g_2},v_0)\ dv_{g_2}dv_0$	$j=j_2, g=g_2$
$\partial\pi_{j_1g_1j_2g_2,y_1,y_2}/\partial\delta_g$	If
$\int_0^1\int_0^1\ f_{Y_{j_2g_2}\|V_{g_2}}(y_{j_2g_2}\|v_{g_2})\ c_{V_{g_2}V_0}(v_{g_2},v_0)\ dv_{g_2}\int_0^1\ f_{Y_{j_1g_1}\|V_{g_1}}(y_{j_1g_1}\|v_{g_1})\ \dot{c}_{V_{g_1}V_0}(v_{g_1},v_0)\ dv_{g_1}dv_0$	$g=g_1$
$\int_0^1\int_0^1\ f_{Y_{j_1g_1}\|V_{g_1}}(y_{j_1g_1}\|v_{g_1})\ c_{V_{g_1}V_0}(v_{g_1},v_0)\ dv_{g_1}\int_0^1\ f_{Y_{j_2g_2}\|V_{g_2}}(y_{j_2g_2}\|v_{g_2})\ \dot{c}_{V_{g_2}V_0}(v_{g_2},v_0)\ dv_{g_2}dv_0$	$g=g_2$
